# Early 2022 breakthrough infection sera from India target the conserved cryptic class 5 epitope to counteract immune escape by SARS-CoV-2 variants

**DOI:** 10.1128/jvi.00051-25

**Published:** 2025-03-26

**Authors:** Indrani Das Jana, Kawkab Kanjo, Subhanita Roy, Munmun Bhasin, Shatarupa Bhattacharya, Indranath Banerjee, Subhasis Jana, Arjun Chatterjee, Alok Kumar Chakrabarti, Suman Chakraborty, Budhaditya Mukherjee, Raghavan Varadarajan, Arindam Mondal

**Affiliations:** 1 Department of Bioscience and Biotechnology, Indian Institute of Technology Kharagpur 30133 , Kharagpur, India; 2 Molecular Biophysics Unit (MBU), Indian Institute of Science 428779 , Bengaluru, India; 3 School of Medical Science and Technology, Indian Institute of Technology Kharagpur 624334 , Kharagpur, India; 4 B.C. Roy Technology Hospital, Indian Institute of Technology Kharagpur 30133 , Kharagpur, India; 5 Purba Medinipur District Hospital, Tamluk, India; 6 Institute of Neuroscience, Kolkata, India; 7 Division of Virology, ICMR-National Institute of Cholera and Enteric Diseases 30170 , Kolkata, India; 8 Department of Mechanical Engineering, Indian Institute of Technology Kharagpur 530004 , Kharagpur, India; University of Kentucky College of Medicine, Lexington, Kentucky, USA

**Keywords:** SARS-CoV-2, spike protein RBD, epitope mapping, breakthrough infection, convalescent serum, yeast display library, neutralizing antibodies, class 5/RBD8 epitopes, immune escape, Omicron subvariant XBB.1.5

## Abstract

During the coronavirus disease 2019 (COVID-19) pandemic, the vast majority of epitope mapping studies have focused on sera from mRNA-vaccinated populations from high-income countries. In contrast, here, we report an analysis of 164 serum samples isolated from patients with breakthrough infection in India during early 2022 who received two doses of the ChAdOx viral vector vaccine. Sera were screened for neutralization breadth against wild-type (WT), Kappa, Delta, and Omicron BA.1 viruses. Three sera with the highest neutralization breadth and potency were selected for epitope mapping, using charged scanning mutagenesis coupled with yeast surface display and next-generation sequencing. The mapped sera primarily targeted the recently identified class 5 cryptic epitope and, to a lesser extent, the class 1 and class 4 epitopes. The class 5 epitope is completely conserved across all severe acute respiratory syndrome coronavirus 2 (SARS-CoV-2) variants and for most sarbecoviruses. Based on these observations, an additional 26 sera were characterized, and all showed a broad neutralizing activity, including against XBB.1.5. This is in contrast with the results obtained with the sera from individuals receiving multiple doses of original and updated mRNA vaccines, where impaired neutralization of XBB and later variants of concern (VOCs) were observed. Our study demonstrates that two doses of the ChAdOx vaccine in a highly exposed population were sufficient to drive substantial neutralization breadth against emerging and upcoming variants of concern. These data highlight the important role of hybrid immunity in conferring broad protection and inform future vaccine strategies to protect against rapidly mutating viruses.

**IMPORTANCE:**

Worldwide implementation of coronavirus disease 2019 (COVID-19) vaccines and the parallel emergence of newer severe acute respiratory syndrome coronavirus 2 (SARS-CoV-2) variants have shaped the humoral immune response in a population-specific manner. While characterizing this immune response is important for monitoring disease progression at the population level, it is also imperative for developing effective countermeasures in the form of novel vaccines and therapeutics. India has implemented the world’s second largest COVID-19 vaccination drive and encountered a large number of post-vaccination “breakthrough” infections. From a cohort of patients with breakthrough infection, we identified individuals whose sera showed broadly neutralizing immunity against different SARS-CoV-2 variants. Interestingly, these sera primarily target a novel cryptic epitope, which was not identified in previous population-level studies conducted in Western countries. This rare cryptic epitope remains conserved across all SARS-CoV-2 variants, including recently emerged ones and for the SARS-like coronaviruses that may cause future outbreaks, thus representing a potential target for future vaccines.

## INTRODUCTION

Severe acute respiratory syndrome coronavirus 2 (SARS-CoV-2) has been circulating in the human population for over 4 years and rapidly evolving, causing the emergence of more than 50 different variants to date (1). Multiple waves of infections were caused by the new variants despite massive vaccination programs, and their emergence was largely driven by the host immunity generated through natural infections and vaccination (2). Detailed molecular-level understanding of the humoral immunity conferred by different vaccination regimens coupled with ongoing viral exposures is important for the development of effective countermeasures against the current and future variants of SARS-CoV-2 and other sarbecoviruses that may emerge in the future.

More than 5.55 billion people, 72.3% of the world population, have received a single dose of coronavirus disease 2019 (COVID-19) vaccine (3), although in Africa, this number is less than 40% (4). India ranks the second highest after China in the number of COVID-19 vaccine doses administered (2.2 billion doses) (5). The vaccination drive started in India on 16 January 2021, at the end of the first wave of infections caused mainly by the circulating B.1.1.7 (Alpha) and B.1.135 (Beta) variants. While the vaccination program was ongoing, the highly infectious B.1.617.2 (Delta) variant emerged and caused the second wave of the pandemic (March to October 2021), which resulted in the highest number of daily infections with high disease severity and mortality rate (6, 7). By January 2022, more than 77% of the Indian population was immunized; the new highly transmissible and heavily mutated B.1.1.529 (Omicron) variant emerged and led to the third wave of infection with milder symptoms and lower fatality rates (8). A significant fraction of the vaccinated population in India got infected during the second and third waves of the pandemic, leading to the large number of “breakthrough infections” (9), which shaped the transmission dynamics and contributed to the overall evolution of the virus (10). After 2021, there was little vaccine uptake in the country, and the percentage of the population that received a third (booster) dose was only about 10%.

The SARS-CoV-2 spike protein (S) is the main surface glycoprotein that mediates the interaction with the host angiotensin-converting enzyme 2 (ACE2) receptor and subsequent fusion of the viral envelope with the host cell membrane that marks the initiation of the infectious cycle (11, 12). The S protein is the primary target for neutralizing antibodies elicited through natural infection as well as vaccination (13–15). The spike constitutes a trimer where individual protomers are comprised of two subunits, S1 and S2 (16). S1 harbors the N-terminal domain (NTD) and the receptor-binding domain (RBD), whereas S2 houses the fusion peptide, the heptad repeats 1 and 2 (HR1, HR2), the transmembrane domain (TM), and the C-terminal domain (CD). The majority of human neutralizing antibodies, isolated from convalescent or vaccinated sera, target conformational epitopes in the RBD receptor-binding motif (RBM) or in the conserved core of RBD (17–19). In the context of the spike trimer, the individual RBDs can be in either the up or down conformation, with only the former able to bind the ACE2 receptor.

The epitopes targeted by neutralizing antibodies can be classified into four major classes (class 1–4) (20). Class 1 and class 2 epitopes overlap with the ACE2 binding site (RBM), and antibodies targeting these sites competitively inhibit receptor binding. While class 1 epitopes are accessible when RBD is present in the up conformation, class 2 epitopes are accessible in both up and down conformations (21). In general, the antibodies targeting class 1 and class 2 epitopes have strong neutralizing activity. However, different SARS-CoV-2 variants escape neutralization by these antibodies by acquiring mutations in the RBM region, compromising their efficacy. Class 3 and class 4 antibodies bind to the RBD core region, away from the ACE2 binding site. N343 glycan and the surrounding residues are accommodated in class 3 epitopes (22). The S309 antibody, a class 3 antibody isolated from a SARS-CoV infected individual, exhibited broad neutralization against SARS-CoV and SARS-CoV-2, although many SARS-CoV-2 variants of concern (VOCs) acquired mutations in class 3 epitopes to escape neutralization (21). The cryptic epitope around the RBD base (first described for CR3022 antibody) constitutes the highly conserved class 4 epitope and is accessible only in the RBD up conformation (23–26). Antibodies targeting class 4 epitopes are suggested to protect against emerging VOCs and other sarbecoviruses. A highly conserved class 5 epitope has been recently identified and characterized as a target for broadly neutralizing antibodies (27, 28).

Mapping conformational epitopes targeted by human polyclonal sera, while challenging, can elucidate how escape mutations in the viral antigen impact the neutralization potency of a heterogeneous mixture of antibodies. This can aid the development of future vaccines and therapeutics. In the present study, we have conducted a comprehensive analysis of human sera collected from individuals vaccinated with two doses of the ChAdOx1 nCoV-19 viral vectored vaccine and who subsequently had breakthrough infections. Neutralization efficacies of 164 serum samples were screened against SARS-CoV-2 pseudoviruses representing the parental strain WA.1 or VOCs B.1.617.1, B.1.617.2, and B.1.1.529. Nine individuals whose serum samples exhibited broader neutralization were subjected to longitudinal sampling, and three of them were selected for epitope mapping due to their high neutralization breadth and potency. Utilizing a charge scanning mutagenesis library (29, 30) of spike RBD, we mapped the epitopes targeted by the polyclonal sera and observed either class 1 epitopes or the rare cryptic class 5 epitope (31) targeted by a major fraction of antibodies. The class 5/ RBD8 epitope residues identified in this study are highly conserved across all SARS-CoV-2 variants, consistent with the observed neutralizing potency of the selected serum samples against heavily mutated Omicron BA.1, BA.5, and the recent XBB.1.5 variant. Based on this observation, an additional 26 sera were characterized and were shown to neutralize XBB.1.5. Our findings illustrate how a combination of vaccination and breakthrough infections in a young, highly exposed population can elicit diverse antibody classes that confer potent neutralizing activity. This may provide insights into the low disease burden that prevailed post-2021 in low- and middle-income countries (LMICs) even in the absence of variant-updated vaccine boosters.

## RESULTS

### Study cohort

**Fig 1:**
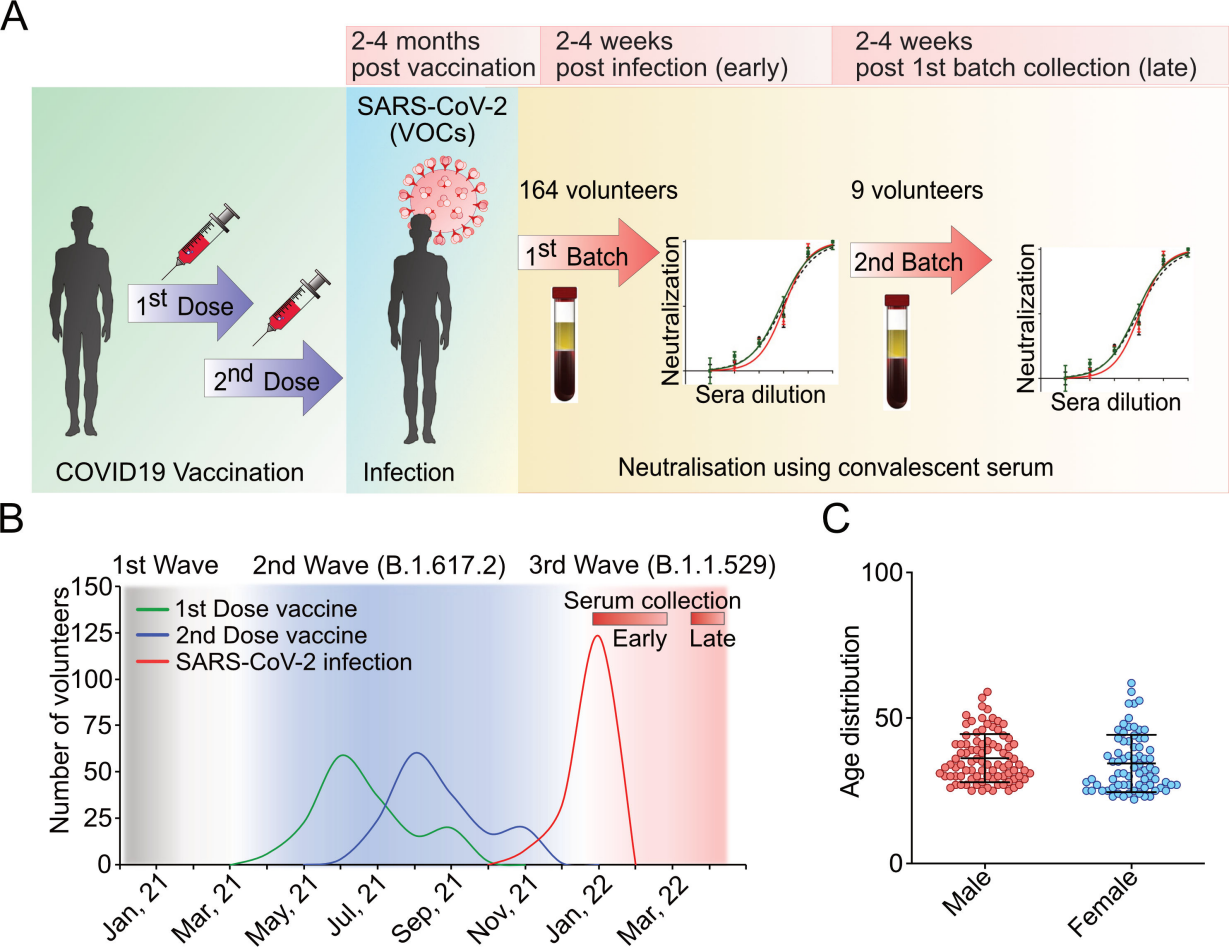
Study cohort and timeline. (A) Schematic representation of study design. (B) Distribution of the study cohort as a function of the timeline of vaccination and breakthrough infection in the context of the second and third waves of pandemics caused by different VOCs of SARS-CoV-2. Red bars indicate the time points of serum sample collection for the entire cohort (early) and subsequently for the selected individuals (late). (C) Age distribution of male and female volunteers included in the study cohort.

In contrast to vaccination alone, hybrid immunity resulting from SARS-CoV-2 breakthrough infections has been shown to boost neutralizing antibody titers while increasing their breadth at the same time (32, 33). We selected individuals who received two doses of the ChAdOx1 nCoV-19 viral vector vaccine between April and November 2021 and then were infected between November 2021 and January 2022, which marks the transition between the second and third waves of the pandemic in India, caused by the B.1.617.2 (Delta) and B.1.1.529 (Omicron) variants, respectively (34) (Fig. 1A and B). We hypothesized that such individuals would have developed broadly neutralizing antibodies that are effective against different variants of concern (32, 33). Blood samples, along with questionnaires about gender, age, and symptoms, were collected from 164 selected individuals who were voluntarily recruited by separate consents for this study. All participants were confirmed positive by RT-PCR for SARS-CoV-2 infection during the abovementioned time frame, which is within 2–4 months post-second dose of vaccination. The serum samples were collected from the individuals within 4–6 weeks of their breakthrough infection (Fig. 1A and B). The study cohort consisted of 90 males and 74 females; the median ages were 36 and 34 years, respectively (Fig. 1C). Most volunteers showed mild symptoms such as fever, head and body ache, cough, chest congestion, and loss of smell with no cases of hospitalization. Only 5.84% (10 individuals) showed severe to critical illness with declining oxygen saturation and breathlessness along with the abovementioned symptoms.

### Neutralization profile of the polyclonal serum samples against SARS-CoV-2 variants

**Fig 2:**
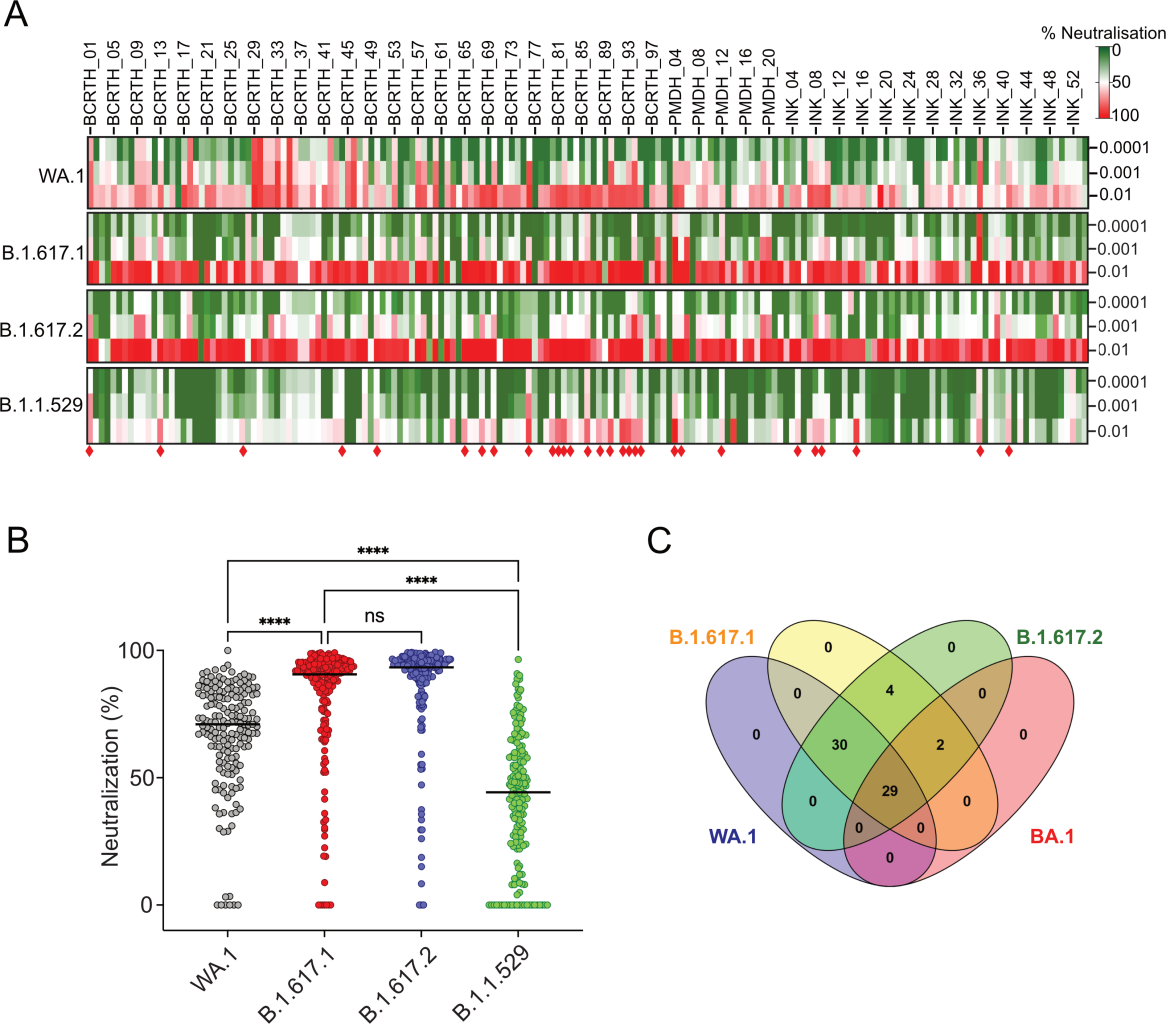
Neutralizing activity of the polyclonal serum samples isolated from the study cohort. (A) Heatmap of neutralizing activity of the 164 serum samples (at dilutions; 0.01, 0.001, 0.001) against the parental strain and different VOCs of SARS-CoV-2, with green to red representing the lowest to highest extent of neutralization (%). (B) Comparison of the relative neuralization efficacies of the serum samples (at 0.01 dilution) against the parental strain and different VOCs. Results were compared by performing two-way ANOVA, and significance is defined as *P* < 0.0001 and denoted as asterisks (****). (C) Venn diagram showing a cross-reactivity of the selected serum samples displaying more than 65% neutralization (at 0.01 dilution) toward at least one of the pseudoviral variants tested in this study. A total of 29 serum samples with >65% neutralization toward all the VOCs tested in this study were designated as broadly neutralizing sera (red diamonds in A). Each image shown here is a representative of three independent experiments performed in triplicate.

We employed a high-throughput neutralization assay based on reporter lentiviruses pseudotyped with spike proteins from SARS-CoV-2 parental WA.1, B.1.617.1 (Kappa), B.1.617.2 (Delta), and B.1.1.529 (Omicron) strains (35). A high degree of correlation between the pseudoviral and live virus-based neutralization assays has been established in several laboratories including our own, which validates the use of pseudoviral neutralization assays in the present work (36–39). Pseudoviruses were incubated with dilutions of serum samples ranging from 10^−2^ to 10^−4^ before infecting HEK-293T cells overexpressing human ACE2. The reporter signal was measured as a proxy of infectivity, which was the highest for the mock-treated virus and the lowest for a polyclonal antibody pre-treatment that binds the spike protein and shows high neutralization activity (Novus Biologicals, NB100-56578). Neutralization efficacies of the 164 serum samples (at three dilutions) against WA.1 and different VOCs were represented as a heatmap with colors ranging from red (high neutralization) to green (low neutralization) (Fig. 2A). Overall, the serum samples from this study exhibited maximum neutralizing activity toward the Delta variant, followed by the Kappa variant with a geometric mean (GM) of 93% and 90%, respectively (Fig. 2B). Reduced neutralizing activity was observed against the parental WA.1 strain (GM 73%), which was further lowered against the Omicron variant (GM 46%) (Fig. 2B). These results suggest that the majority of the individuals in the study cohort had breakthrough infections caused by the Delta variant. Most of the serum samples neutralized Delta and Kappa variants to similar extents (Fig. 2A and B), likely due to the high similarity between the spikes of both variants (40). A few serum samples showed high neutralization activity against the Omicron variant (Fig. 2A and B). Limited neutralization activity against the Omicron variant results from the high number of mutations in this variant, conferring extensive immune escape from vaccine and infection sera (41).

### Breadth and cross-reactivity of the selected polyclonal serum

In the initial screening, 29 of the 164 breakthrough infection sera (17%) showed broadly cross-reactive neutralizing activity (>65% neutralization) against WA.1, Kappa, Delta, and Omicron variants at 10^−2^ dilution (Fig. 2A and C, red diamonds); and hence were considered as broadly neutralizing serum samples. We tracked these 29 individuals and selected nine volunteers who had not been infected and had not received any vaccine booster after the first round of blood collection. Subsequently, a second set of serum samples (designated “late”) was collected from the nine selected individuals with proper consent, 4–6 weeks after the first set (designated “early”) (Fig. 1A and B), and both early and late sets of the serum samples were subjected to further analysis.

**Fig 3:**
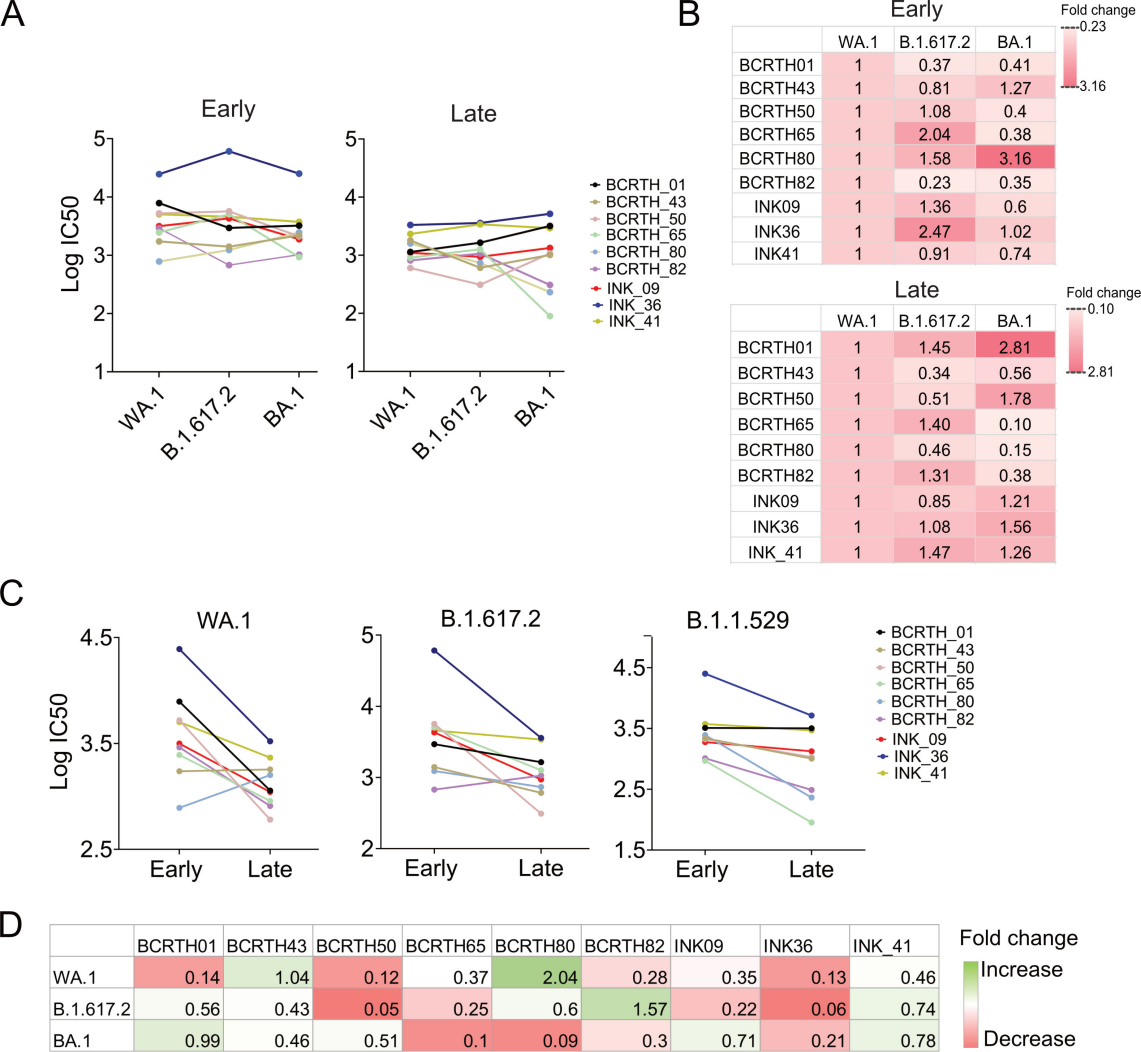
Comparative neutralizing activities of the selected serum samples collected at early and late time points. (A) Comparative analysis of the neutralization titers (IC50) of serum samples isolated from nine selected volunteers during the first (early) and second (late) batches of sample collection, against WA.1, B.1.617.2, and BA.1. (B) Heatmap showing the fold change in the IC50 values for B.1.617.2 and BA.1 w.r.t. the parental WA.1 strain, with red to white indicating higher to lower neutralization titers. (C) Comparative analysis of the pseudoviral neutralization titers of the serum samples isolated at early and late time points. (D) Heatmap representing the variant-specific fold change in IC50 values. All data shown are averages of the results of at least three independent experiments.

Neutralization efficacies of the selected serum samples were determined against WA.1, Delta, and Omicron variants using the pseudovirus neutralization assay (Fig. S1A and B), and neutralization titers were represented in the form of IC50 (Fig. 3A and B). Among the nine serum samples collected at the early time point, four showed the highest IC50 values for Delta, three for the parental WA.1 strain, and two for the Omicron; a trend that matched with the overall neutralizing activity of the larger cohort of 164 samples (Delta > WA.1 > Omicron; Fig. 2B). However, this was not the case for the serum samples collected from the same volunteers at the later time point (Fig. 3A and B). A moderate to sharp decline in neutralizing activity (2- to 18-fold change in IC50) was observed for most serum samples between the early and late time points (Fig. 3C and D). A few sera had an increase or maintained similar variant-specific neutralization titers at both time points. Compared to the early time point neutralization profile, BCRTH-01 and INK-41 showed a significant decline in neutralization titer against WA.1 but a slight increase in neutralization titer against the Omicron variant at the late time point (Fig. 3C and D). This may reflect the higher durability of the variant-specific (Omicron or Delta) antibodies generated by the breakthrough infections with respect to (w.r.t.) the parental strain (WA.1) specific antibodies elicited through vaccination. Interestingly, BCRTH-01, INK-09, INK-36, and INK-41 sera maintained high neutralization titers against all the pseudoviruses tested and at both time points, with INK-36 being the most potent neutralizing serum (Fig. 3A through C).

Apart from virus neutralization, antibody-dependent cytotoxicity (ADCC) and other effector functions are key immune responses that confer protection against virus infection by selective recognition of the infected cells by specific antibodies, followed by destruction of these cells by natural killer (NK) cells. To assess the potential of the selected polyclonal sera to confer ADCC, we used Raji cells, a Burkitt lymphoma cell line known to resist NK cell-mediated killing under normal conditions. Raji cells expressing SARS-CoV-2 spike protein through transient transfection were treated with either BCRTH-01 sera or control sera and subsequently co-incubated with peripheral blood mononuclear cells (PBMCs) (isolated from a healthy donor) as source of NK cells. Compared to the pre-COVID-19 era control sera, treatment with BCRTH01 sera resulted in a significant reduction in the Raji cell population, indicative of antibody-dependent cytotoxicity (Fig. S2). This finding was corroborated by a significant increase in the activation of CD107a+ NK cells within the PBMC population, in response to BCRTH01 sera compared to control sera (Fig. S4). Notably, untransfected Raji cells incubated with BCRTH-01 sera displayed similar percentages of Raji cells and activated NK cells as those treated with control sera, highlighting the spike-specific nature of the cytotoxic response elicited by BCRTH-01 sera. These data indicate that at least some of the sera selected in this study not only have broad neutralizing activity but also are potent in clearing infection through ADCC.

**Fig 4:**
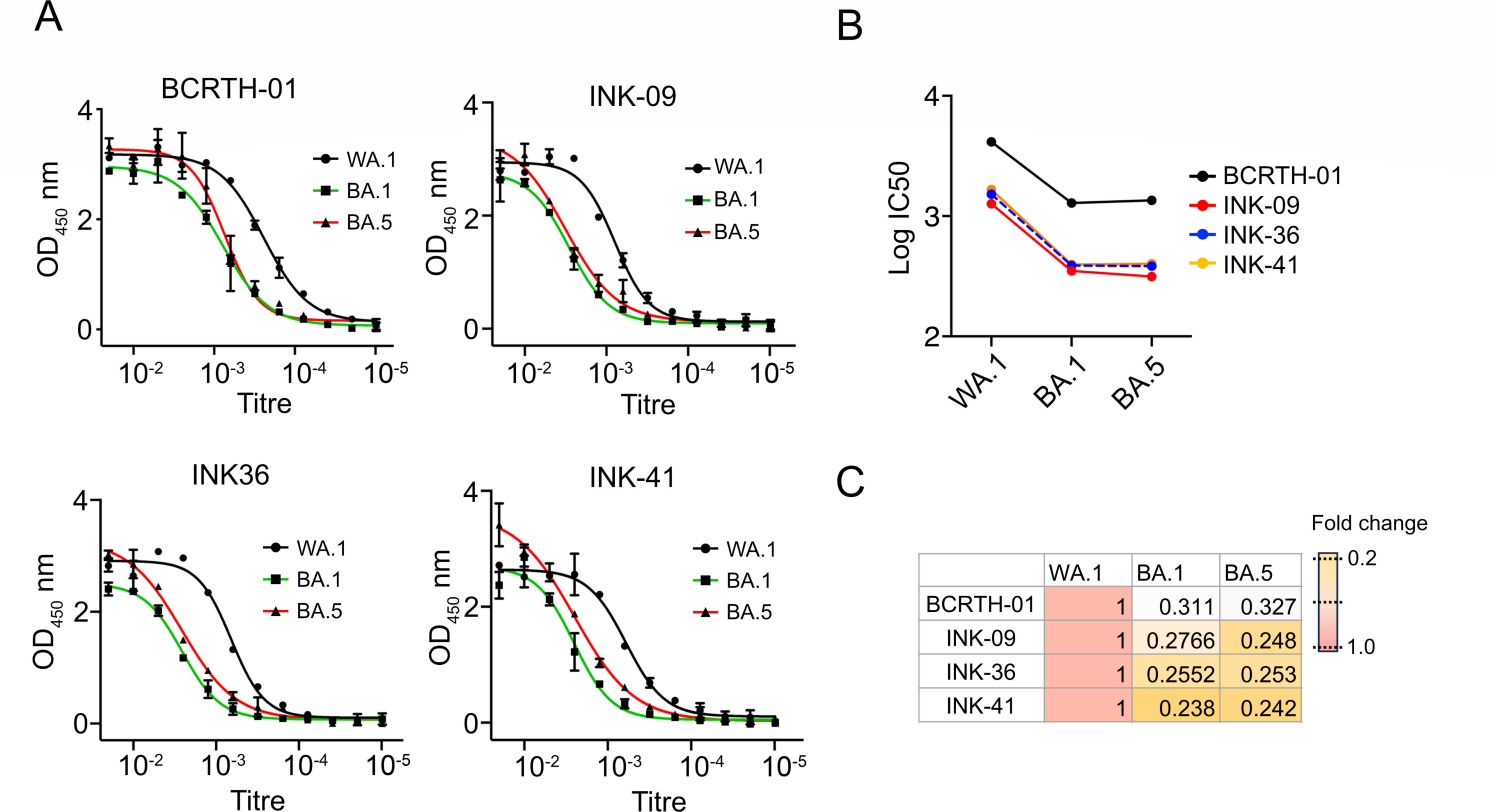
The serum samples show high binding affinity toward spike RBD. (A) ELISA was performed to determine the binding affinity of the polyclonal serum samples toward spike RBD derived from the WA.1 and Omicron subvariants BA.1 and BA.5. (B) Comparison of the binding affinity of different serum samples (presented in the form of IC50) toward different RBD variants. (C) Fold change in IC50 values for the BA.1 and BA.5 variants w.r.t. the WA.1. Each data shown is a representative of at least three experiments performed independently.

The majority of the neutralizing antibodies target the RBD of the spike protein (17–19). Hence, we sought to test the binding activity of the four serum samples (BCRTH-01, INK-09, INK-36, and INK-41) to the spike RBD. Enzyme-linked immunosorbent assay (ELISA) was performed with recombinant purified spike RBD derived from the WA.1, Omicron BA.1, and BA.5 isolates. These were used as coating antigens. As expected, all four serum samples bound well to the RBD, with the BCRTH-01 sera showing the highest binding affinity for all three RBD variants. This suggests a high abundance of RBD-targeted antibodies in the selected convalescent serum (Fig. 4A). In general, the serum samples showed the highest IgG titers against WA.1 and marginally lower titers against BA.1 and BA.5 (3- to 4.2-fold reduction) (Fig. 4B and C). As all four serum samples showed comparable binding titers against the RBD of WA.1, BA.1, and BA.5, we selected three of them, namely BCRTH-01, INK-09, and INK-36, for epitope mapping study.

### Comprehensive mapping of RBD epitopes targeted by the selected human sera

**Fig 5:**
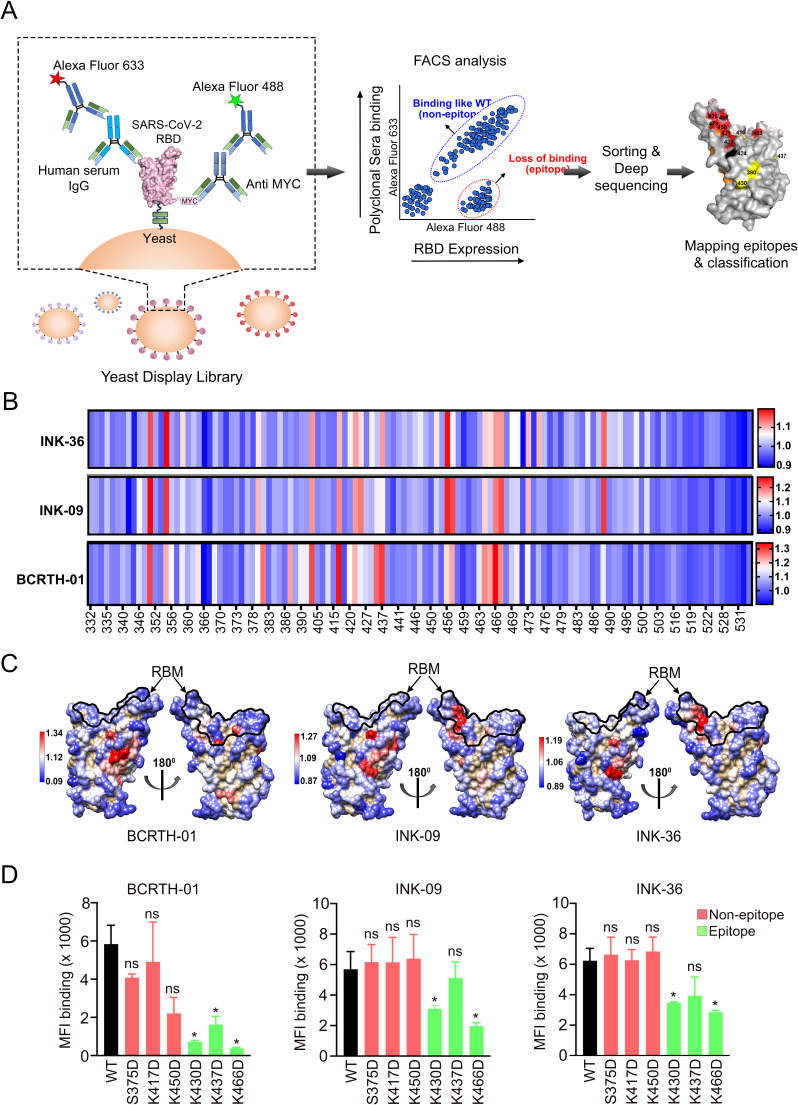
Identification and validation of RBD epitope residues through yeast display analysis. (A) Schematic representation of the yeast display screening. (B) The heatmap represents the ratio of MFI expression to MFI binding extracted from analysis of deep sequencing reads. The higher to lower ratios are represented in a red to blue gradient. The higher the ratio (red), the higher the extent of serum escape at that position. The epitope mapping study was performed in duplicate. (C) The MFI expression/MFI binding ratios are mapped on the RBD structure (PDB ID: 6M0J) following the same color scheme. The RBM is demarcated on the structure using a black line. (D) Validation was carried out using FACS by quantifying the binding of WT and the mutant RBDs, harboring mutations at the epitope and non-epitope residues, to BCRTH-01, INK09, and INK36 serum samples. The MFI binding of WT and mutant RBDs w.r.t. the three serum samples is presented. Each image is representative of two independent experiments performed in triplicate. Results were compared by performing a one-way ANOVA, and significance is defined as *P* < 0.01 and denoted as asterisk (*).

We employed a charged scanning mutagenesis library of SARS-CoV-2 RBD displayed on the yeast surface as a novel approach to identify the epitopes targeted by the antibodies present in the polyclonal serum samples (Fig. 5A). WA.1 RBD (332-532) was used as the template for library construction, and all RBD residues with a solvent accessibility >15% were individually mutated to negatively charged Aspartate, with the exception of negatively charged residues, which were mutated to the positively charged Arginine residue. The generated RBD library was displayed on the yeast surface and subjected to binding with a saturating concentration of the polyclonal serum sample as determined by sera titration with the WT RBD displayed on the surface of the yeast cells (Fig. S3A). Binding was detected using fluorescently labeled antibodies, and the yeast cell populations were sorted into different bins through fluorescence-activated cell sorting (FACS) (Fig. S3B through D). Yeast populations within these bins were subjected to deep sequencing to identify the mutations responsible for abolishing binding to the serum. The deep sequencing reads were analyzed as described in the methods section. Briefly, the binding and expression signals of each mutant RBD were normalized with with respect to the wild type, and the ratio between the normalized mean fluorescence intensities for expression (MFI expression) and binding (MFI binding) for each of the RBD variants was calculated (Table S1).

Epitope mapping was performed with all three of the selected serum samples (BCRTH-01, INK-09, or INK-36). RBD residues whose mutation resulted in MFI_Ratio_ of one standard deviation (SD) higher than the mean MFI_Ratio_ were considered epitope residues (Fig. 5B; Table 1). A high value of MFI_Ratio_ (darker shades of red) indicates that the charged substitution significantly perturbed the binding of the serum samples with minimum or no effect on expression. The MFI_Ratio_ of the identified epitope residues was mapped on the RBD structure (Fig. 5C). Overall, the identified epitopes (highlighted in lighter to dark shades of red) for all three serum samples were clustered in two major groups: (i) epitopes that overlap with the ACE2 binding motif (RBM designated by a black line on the RBD structure, Fig. 5C) and (ii) epitopes that are away from the RBM. Interestingly, for BCRTH-01 serum, an enrichment in non-RBM over RBM epitope residues was observed. However, this was not the case for INK-36 or INK-09, where epitope residues across different RBD regions were enriched to similar levels (Fig. 5C).

**TABLE 1:** MFI ratios for the potential epitope residues^*a*^

Sl no.	Residue	MFI expression/MFI binding		
		BCRTH-01	INK-09	INK-36
1	351	1.295788	1.273718	1.157062
2	355	1.219729	1.239262	1.197349
3	356	1.17089	–	–
4	359	–	–	1.081459
5	364	1.182295	–	–
6	380	–	1.148814	1.077429
7	381	1.260786	–	–
8	387	1.230637	–	–
9	403	1.292267	1.172926	1.120017
10	416	1.329536	1.175896	1.101252
11	420	1.163761	–	1.072276
12	421	1.218064	1.168982	1.115263
13	424	–	1.192174	1.081695
14	430	1.270088	–	–
15	437	1.301646	1.107961	1.117455
16	452	–	–	1.076026
17	456	1.185845	1.252503	1.192579
18	457	1.225249	1.222689	–
19	464	1.246659	1.128143	1.087599
20	465	1.225858	–	1.080284
21	466	1.341557	1.187018	1.116536
22	467	1.242733	1.221766	1.118558
23	470	1.178698	–	1.063863
24	473	1.169721	1.145745	1.100281
25	475	–	–	1.102217
26	489	–	1.234014	1.144957

^*a*^ – signifies that the residue is not identified in the analysis as an epitope residue for this serum sample.

To this end, we aimed to validate the results of the yeast-display screening and deep sequencing by individually mutating a few of the epitope and non-epitope residues identified in our analysis and evaluating their impact upon serum binding. Considering that validating all of the library residues would be difficult, we selected three epitope and three non-epitope residues that are identified in the context of all three serum samples (BCRTH-01, INK-09, and INK-36) and reintroduced these mutations individually into RBD. Subsequently, the extent of binding of these mutant RBDs by all three serum samples was evaluated by yeast surface display followed by flow cytometry (Fig. S4A through C). We selected the non-RBM epitope residues (T430, T437, and R466) as they showed high values of MFI_Ratio_ compared to the wild type. As shown, charged substitution at the putative epitope residues significantly impacted the binding to the serum samples while charged substitution at putative non-epitope residues had no effect upon the binding (Fig. 5D). The impact of mutating the epitope residues was most prominent for BCRTH-01, possibly because the highest abundance of RBD-specific antibodies was observed in the BCRTH-01 serum (Fig. 4B). These data validate the epitope residues identified through the current study and establish the robustness of the charged scanning mutagenesis approach for the identification of epitope residues targeted by polyclonal human serum.

### Antibodies in the selected sera target a cryptic epitope that is broadly conserved across different SARS-CoV-2 variants

**Fig 6:**
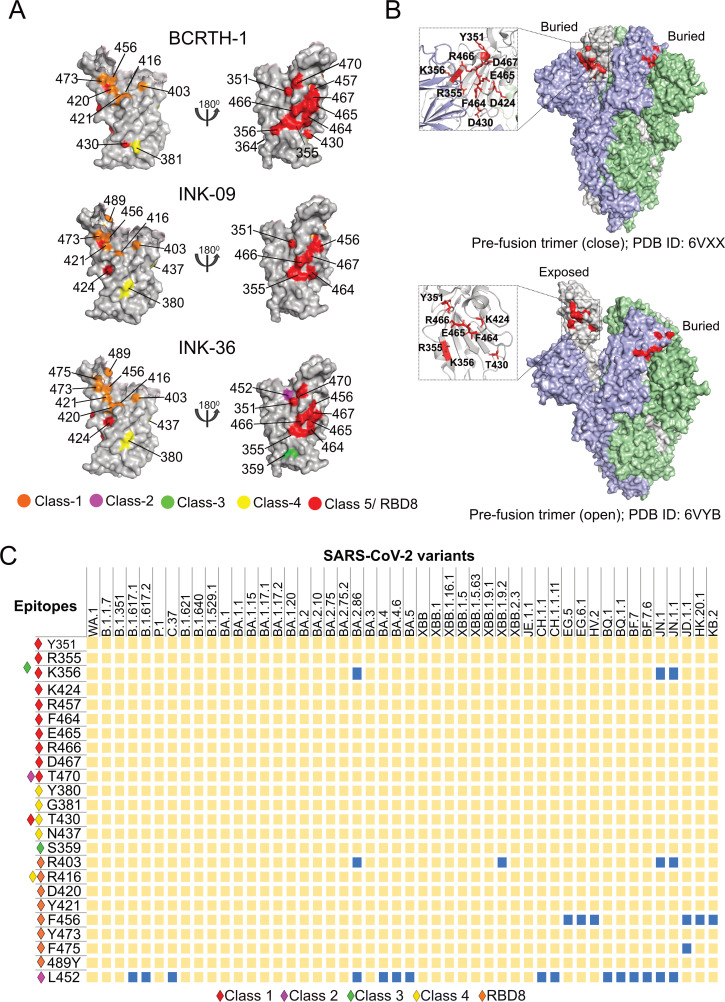
Epitope classification and characterization. (A) Individual epitope residues, as identified for the BCRTH-01, INK-09, and INK-36 serum samples, are mapped on the surface of the RBD (PDB ID: 6M0J) and are color coded based on the previously published epitope classes. (B) Class 5/RBD8 epitopes identified in this study are mapped on the pre-fusion trimeric structure of the spike in its closed (PDB ID: 6VXX) and open (PDB ID: 6VYB) conformations. (C) Matrix representing the conservation of identified epitope residues across all major SARS-CoV-2 variants. Mutations of a specific residue in a particular variant are represented in blue squares. Epitope classes are designated using colored diamonds.

Previous epitope mapping studies on convalescent polyclonal serum samples showed enrichment of antibodies targeting the class 1, 2, and 3 epitopes and to a lesser extent the class 4 epitope (41–45). Hence, we analyzed the different epitopes identified in the present study and compared them to the epitopes reported in the literature. The majority of the neutralizing epitope residues in the RBD are categorized into four major classes, class 1–4 (Fig. 6A; Table 2). Out of the 26 epitope residues identified, eight were classified as class 1, two as class 2, two as class 3, and five as class 4. Surprisingly, 11 out of the 26 (42%) epitope residues do not belong to any of these major classes (except for K356 which is shared with class 3 and T470 which is shared with class 2), but instead belong to a recently identified rare cryptic epitope (46, 47), situated away from the RBM (Fig. 6A) and designated as class 5 or RBD8 class epitope (31).

**TABLE 2:** Classification of epitopes^*a*^

Residue no.	Epitopes for serum samples			Epitope class	Antibody	Reference
	BCRTH-01	INK-09	INK -36			
351	+	+	+	Class 5/RBD8	N-612–056; BIOLS56/IMCAS74/WRAIR-2057/WRAIR-2134	(31, 46–48)
355	+	+	+	Class 5/RBD8	N-612–056/BIOLS56/IMCAS74/WRAIR-2063/S2H97/FD20/WRAIR-2057/WRAIR-2134	(27, 31, 46–50)
356	+	−	−	Class 3/Class 5/RBD8	S309/N-612–056/IMCAS74/WRAIR-2134	(22, 31, 46, 48)
359	−	−	+	Class 3	S309	(22)
364	+	−	−	Unclassified	−	−
380	−	+	+	Class 4	COVA1-16, CR3022	(25, 51)
381	+	−	−	Class 4	COVA1-16, CR3022	(25, 51)
387	+	−	−	Unclassified		−
403	+	+	+	Class 1	C002, C102, C121	(21)
416	+	+	+	Class 1/Class 4	C105, COVA1-16, C118, C022	(25, 26, 52)
420	+	−	+	Class 1	C105, LY-CoV-016	(42, 52, 53)
421	+	+	+	Class 1	C102	(21)
424	−	+	+	Class 5/RBD8	BIOLS56/IMCAS74/WRAIR-2057/WRAIR-2134	(31, 48)
430	+	−	−	Class 4/Class 5/RBD8	C022/BIOLS56/IMCAS74/WRAIR-2057/WRAIR-2134	(26, 31, 48)
437	+	+	+	Class 4	C118	(26)
452	−	−	+	Class 2	C144, C002	(21)
456	+	+	+	Class 1	C002, C102, C121	(21)
457	+	+	−	Class 5	N-612–056, 6D6; WRAIR-2057/WRAIR-2134	(46, 48)
464	+	+	+	Class 5/RBD8	N-612–056/6D6 & 7D6/BIOLS56/IMCAS74/WRAIR-2063/S2H97/WRAIR-2057/WRAIR-2134	(27, 31, 46–50)
465	+	−	+	Class 5/RBD8	N-612–056/6D6 & 7D6/BIOLS56/IMCAS74/WRAIR-2063/S2H97/WRAIR-2057/WRAIR-2134	(27, 31, 46–48, 50)
466	+	+	+	Class 5/RBD8	N-612–056/6D6 & 7D6/BIOLS56/IMCAS74/WRAIR-2063/S2H97/WRAIR-2057/WRAIR-2134	(31, 46, 47)
467	+	+	+	Class 5/RBD8	N-612–056; IMCAS74/WRAIR-2063/S2H97/WRAIR-2057/WRAIR-2134	(31, 46)
470	+	−	+	Class 2/Class 5/RBD8	C144/7D6, 6D6/WRAIR-2057	(21, 47)
473	+	+	+	Class 1	C102	(21)
475	−	−	+	Class 1	C102	(21)
489	−	+	+	Class 1	C102	(21)

^*a*^ + signifies that a particular residue is identified as an epitope for a specific serum sample, while − signifies that it was not.

The class 5 or RBD8 epitope spans diagonally across the base of the RBD (Fig. 6A) and is situated on the opposite surface of the ACE2 binding motif, thereby remaining buried and inaccessible in the prefusion closed conformation of the spike trimer (Fig. 6B). Hence, the binding of class 5 antibodies, such as BIOLS56, IMCAS74, WRAIR-2063/S2H97/WRAIR-2057/WRAIR-2134, to this epitope requires extensive opening of the pre-fusion structure, thereby inducing rapid and premature transition to the post-fusion conformation and S1 shedding (27, 31, 48, 54). These antibodies cross-reacted with all SARS-CoV-2 VOCs, including Delta, Omicron BA.4/BA.5, BQ.1.1, and the recent highly immune-escaping variant XBB1 (31, 48). Supporting this notion, class 5 epitope residues identified in our analysis were highly conserved across all the different SARS-CoV-2 VOCs (Fig. 6C) and across different sarbecoviruses (Fig. S5). All these data together highlight the class 5 epitope as an important target for broadly protective pan sarbecovirus vaccine candidates. Interestingly, the BCRTH-01 serum had the highest number (total 10) of class 5 identified epitope residues compared to INK-09 and INK-36 (7 and 8 residues, respectively) (Fig. 6A; Table 2), which explains its breadth and high cross-reactivity against the different SARS-CoV-2 variants tested. In contrast, INK-36 has the highest number of class 1 and class 2 epitopes (8 and 2, respectively) that are targeted by ACE2 competing antibodies, consistent with it having the highest neutralization activity among the selected serum samples.

To evaluate the effect of charged substitutions on the neutralizing activity of the polyclonal sera, we selected four of the identified RBD-epitope residues and individually mutated them to charged substitutions in the WA.1 backbone to generate mutant pseudoviruses. Two mutations, R403D and G416D, are situated within the RBM and are expected to perturb class 1 or class 1/4 epitope-specific antibodies, respectively, while the other two mutations, T430D and R466D, are situated away from the RBM. T430D is expected to interfere with the binding of class 4 and class 5/RBD8 epitope-specific antibodies, while R466D is expected to exclusively disturb the binding of class 5/RBD8 antibodies (Fig. 6C; Table 2).

**Fig 7:**
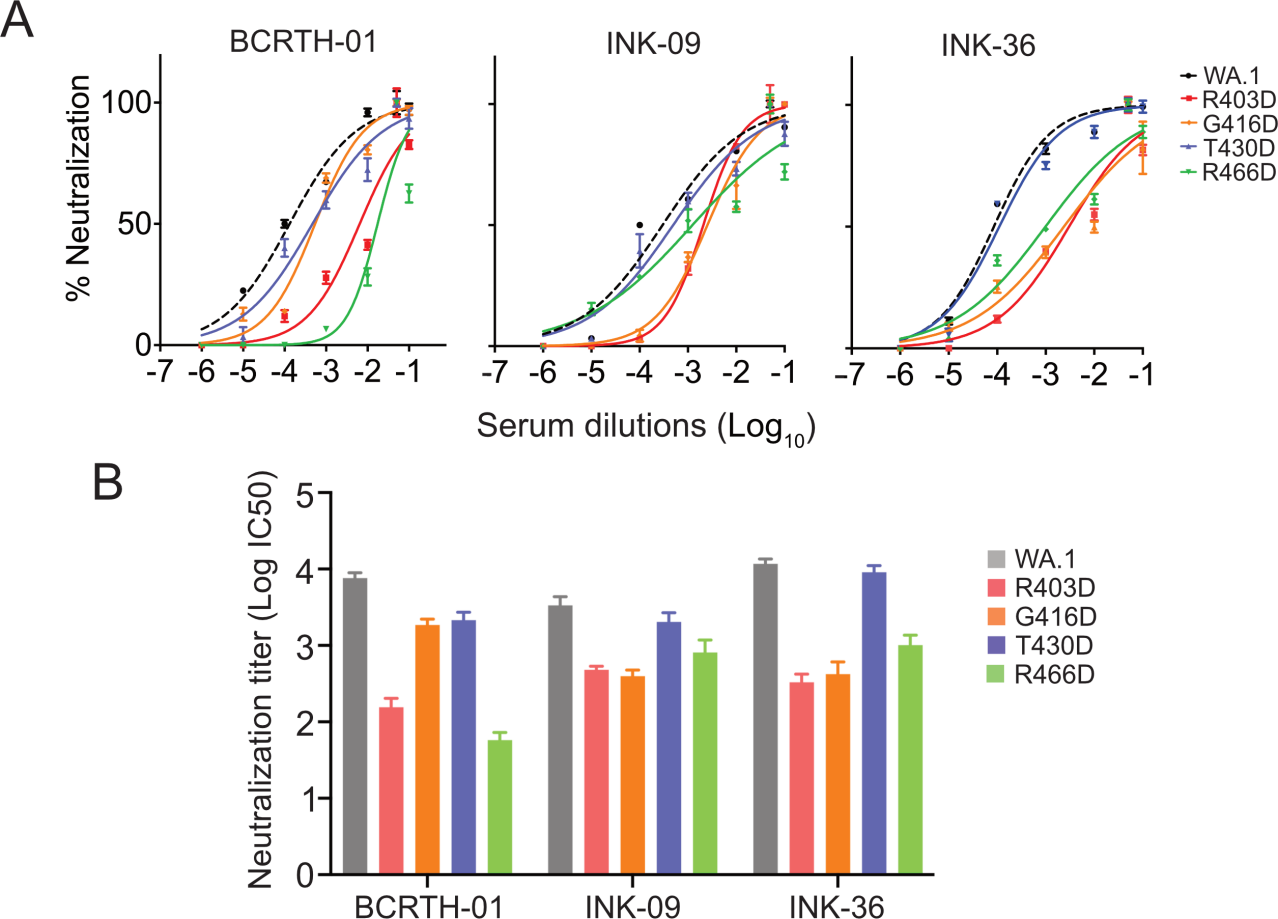
Neutralization titers (IC50) for WT and mutant pseudoviruses w.r.t. selected serum samples. (A) Neutralization curves for pseudoviruses with WT (WA.1) or mutant spike proteins, harboring specific mutations at the epitope residues, with serial dilutions of the BCRTH-01, INK-09, and INK-36 sera as indicated. (B) Neutralization titers (IC50) for WT and mutant pseudoviruses for three sera samples as calculated from (A). The neutralization experiments are performed in three independent biological replicates.

We tested the sensitivity of WT and mutant pseudoviruses to neutralization by BCRTH-01, INK-09, and INK-36 sera (Fig. 7A). As expected, all of the mutations reduced the neutralization activity of all three polyclonal serum samples in comparison to the WT pseudovirus. BCRTH-01 serum showed the highest sensitivity toward R466D and R403D mutations, with a drastic 132-fold and 48.9-fold reduction in the neutralization titer (IC50), respectively (Fig. 7B). In contrast, T430D and G416D mutations showed moderate and comparable effects with 3.55- and 4.12-fold reduction in IC50 values, respectively. On the other hand, INK36 serum showed a high 35.5-fold and 27.8-fold reduction toward R403D and G416D mutations, moderate 11.5-fold reduction toward R466D mutation, and negligible 1.3-fold reduction for T430D mutation, respectively. INK-09 showed the same trend as INK-36 but with less prominent reductions toward the abovementioned mutations. It is to be noted that the T430 residue has been identified as an epitope residue for BCRTH-01 but not for the INK-09 or INK-36 serum samples. Consistent with this, mutations showed negligible reduction toward the neutralization titers of these two sera. Together, our data suggest that the BCRTH-01 serum sample harbors the highest fraction of class 5/RBD8 and class 1 antibodies, while the INK-36 and INK-09 possess a higher fraction of class 1 and class 4 antibodies and lower fraction of the class 5/RBD8 antibodies. This combination of ACE2 competing (class 1) and non-competing (class 5/RBD8 and class 4) antibodies imparts high neutralization efficacy and broad cross-reactivity to all of these serum samples, which render it effective against different variants of SARS-CoV-2, including the currently circulating ones.

### Pre-XBB serum neutralizes the recent Omicron subvariant XBB.1.5

**Fig 8:**
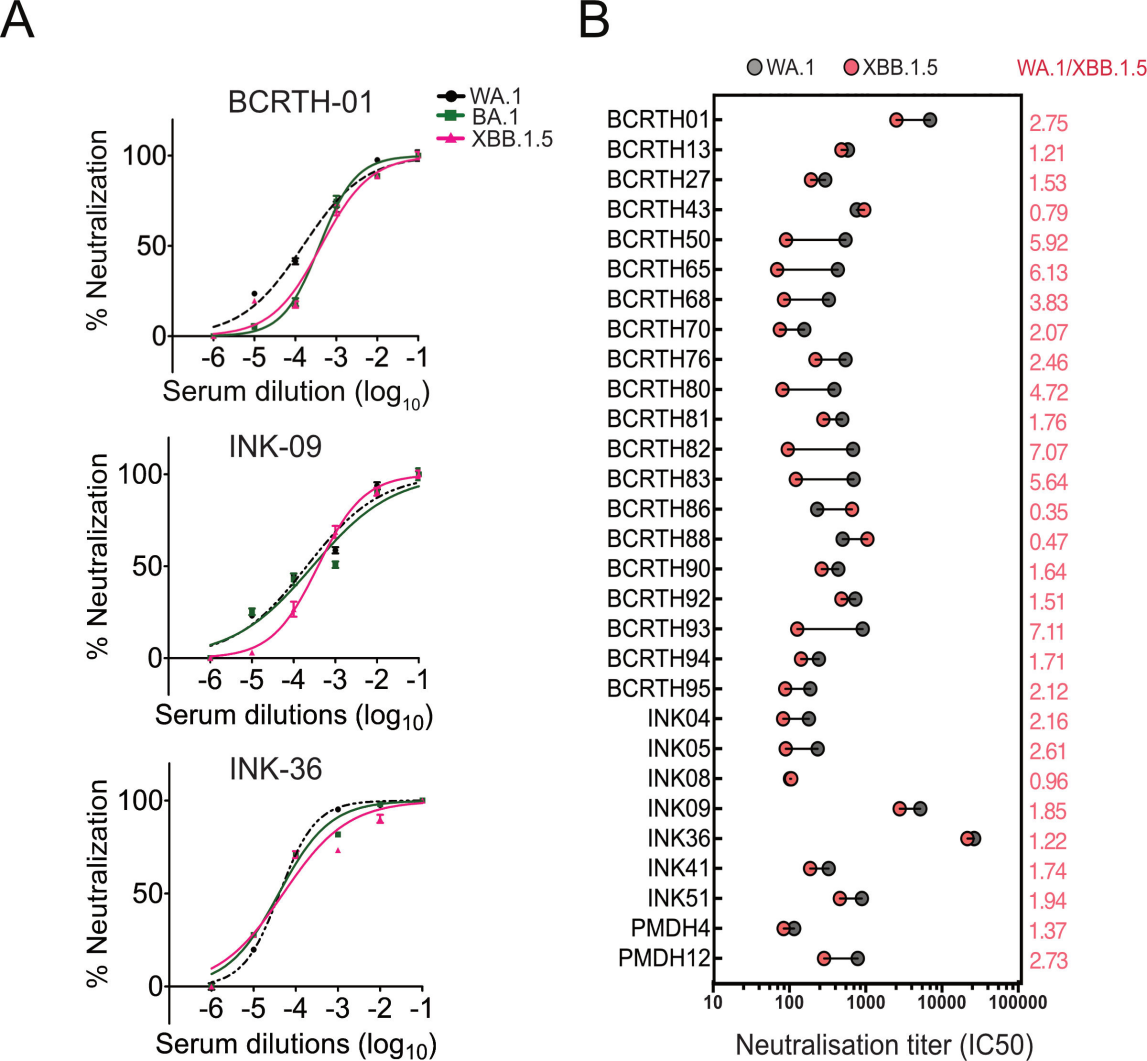
A considerable fraction of the study cohort shows neutralization activity against Omicron XBB.1.5. (A) Neutralization curves for WA.1, BA.1, and XBB.1.5 pseudoviruses with serial dilutions of BCRTH-01, INK-09, and INK-36 sera. The data shown are averages of the results of at least three independent experiments ± SD. (B) Neutralization titers (IC50) of 29 serum samples (from Fig. 2C) w.r.t. WA.1 and XBB.1.5 pseudoviruses as calculated from the neutralization curves shown in Fig. S6. Fold change in the IC50 values for XBB.1.5 w.r.t. the WA.1 are marked in salmon red.

The heavily mutated Omicron subvariants, XBB and XBB.1, emerged in late 2022, and soon, XBB and its descendants accounted for the majority of infections worldwide (55, 56). The variant XBB.1.5 harbors a rare mutation, F486P, which demonstrates superior transmissibility and immune escape toward vaccines and monoclonal antibodies, accounting for its rapid spread in more than 100 countries (57–62). Interestingly, the epitopes identified in this study remain completely conserved for XBB.1.5 (Fig. 6C), suggesting that the selected serum samples should neutralize this more recent subvariant of SARS-CoV-2. Thus, we generated pseudoviruses harboring either WA.1, Omicron BA.1, or Omicron XBB.1.5 spike proteins at their surface and performed neutralization assays with the BCRTH-01, INK-36, and INK-09 serum samples, which were collected in early 2022, well before the emergence of XBB. Interestingly, all three serum samples showed neutralizing activities against Omicron BA.1 and XBB.1.5, comparable to the parental WA.1 strain (Fig. 8A).

It is possible that the XBB.1.5 neutralization activity shown by the three selected serum samples may be an outcome of random stochasticity and does not represent a general trend of the larger study cohort. To investigate this, we evaluated the neutralizing activity of the 29 serum samples, which showed broader cross-reactivity against all of the tested SARS-CoV-2 variants in the initial screen (Fig. 2C). Neutralization curves for the sera against WA.1 or XBB.1.5 pseudoviruses are shown in Fig. S6, and the respective neutralization titers are shown in Fig. 8B. All of the above sera showed comparable neutralizing activity toward WA.1 and XBB.1.5 with a maximum of sevenfold reduction for the latter. These data indicate that a significant fraction (at least 17%) of the participating volunteers, who had breakthrough infections following vaccination during the Delta/Omicron wave (December 2021) of the pandemic in India, generated humoral immunity that remained effective even with subvariants that emerged recently (February–March 2023). This, in turn, establishes the potential importance of the identified epitopes as a target for developing vaccine candidates against future SARS-CoV-2 variants.

## DISCUSSION

Hybrid immunity generated by breakthrough infections has been reported to induce enduring humoral immunity and generate broadly neutralizing antibodies (33, 63, 64) against SARS-CoV-2 variants through several different mechanisms (65). First, vaccination and infection select different B cell clones leading to the generation of broadly cross-reactive neutralizing antibodies (66, 67). Second, the antibodies generated through the primary antigen response directly modulate the secondary response against SARS-CoV-2 by blocking or enhancing the naïve B cell recruitment (68). Third, timely spaced repeated antigen contact through vaccination and subsequent infection or exposure promotes extensive germinal center reactions, resulting in high-affinity cross-reactive neutralizing antibodies (69, 70). These together explain why breakthrough infections and extensive viral exposure activate memory B cells that recognize conserved cross-variant epitopes rather than promoting naïve B cell recruitment (71, 72). In this study, we examined the effects of hybrid immunity in eliciting antibody breadth and characterized the epitopes targeted by the antibodies generated in a cohort of patients with breakthrough infection from India.

Human high-resolution epitope mapping studies following vaccination and/or infection have been largely focused on sera from the US or European populations that were primarily immunized with mRNA vaccines (19, 45, 73, 74). In contrast, a few studies focused on sera from individuals vaccinated with CoronaVac whole-virus inactivated vaccine, with or without breakthrough infection (61, 75, 76). Little is known about the humoral response in India and China, where a large fraction of the population received viral vector or inactivated virus vaccines. Studies have demonstrated substantial escape of lately emerged Omicron variants from the sera of vaccinees immunized with bivalent (WA1/BA.5) mRNA-LNP vaccines (77, 78). Although breakthrough infections, as well as administration of multiple variant-matched boosters of mRNA vaccines, have elevated neutralizing activity toward the recent Omicron variant (XBB), the titers remain considerably lower compared to those against WA.1 (79). Relatively fewer studies have been performed in ChAdOx1 vaccinees for their immune responses to Omicron lineage variants. Individuals from South Africa with prior SARS-CoV-2 infection followed by vaccination with ChAdOx1 showed 10-fold lower neutralization titers against Omicron subvariants BA.1 and BA.4 compared to WA.1 (80). A recent study with two groups of Indian individuals vaccinated with two or three doses of the ChAdOx1 vaccine and infected with SARS-CoV-2 at least once showed a 10-fold reduction in neutralizing activity against the XBB variant, 6 months postvaccination (81). No high-resolution epitope mapping studies have been conducted so far with those receiving exclusively the ChAdOx1 vaccine.

In the present study, we describe epitope mapping of sera from an Indian population where participating volunteers received two doses of ChAdOx1 viral vector vaccine and had a “breakthrough infection” within 4–6 weeks post-second dose. The timing of the study was deliberately chosen at the intersection of the second and third waves of the pandemic instigated by the B.1.617.2 (Delta) and B.1.1.529 (Omicron) variants, which, in theory, were expected to enhance the breadth and potency of antibodies generated through vaccination (parental strain WA.1) and subsequent infections by different VOCs (33). Around 18% (29) of the serum samples exhibited >65% neutralizing activity against the parental strain and different VOCs including Kappa, Delta, and Omicron (Fig. 2C) and showed comparable WA.1 and XBB.1.5 neutralizing titers (≤7-fold reduction for XBB.1.5) (Fig. 8B). These data emphasize the role of hybrid immunity in generating highly potent, durable, and broadly cross-reactive neutralizing antibodies.

We employed an aspartate scanning mutagenesis and yeast surface display coupled with flow cytometry and deep sequencing to map epitope residues, which, upon charged substitution, severely impacted the binding of the serum antibodies without affecting the expression. Charge scanning mutagenesis has been shown to be effective in identifying residues buried at the protein–protein interaction interface (82, 83). While deep mutational scanning has been previously employed to identify serum escape mutations on SARS-CoV-2 RBD (19, 84), the relatively smaller size of the aspartate scanning library, together with the unique barcoding strategy of the individual mutant’s open reading frames (ORFs) employed by us, simplifies library construction and downstream analysis. Using this method, we identified a total of 26 surface-exposed amino acid residues as potential epitopes targeted by the three selected sera. The majority of these residues have been previously identified as part of epitopes for different classes of RBD neutralizing monoclonal antibodies (85), thus supporting the robustness of the novel screening approach in identifying epitopes targeted by polyclonal sera.

The epitopes identified using the proposed analysis can be broadly categorized into RBM epitopes and non-RBM epitopes. The RBM epitopes consist primarily of class 1 and class 2 epitopes, which are targeted by antibodies that competitively hinder receptor engagement and virus attachment to host cells. While antibodies targeting these epitopes are responsible for the high neutralizing activity of polyclonal serum used in this study (17, 21, 86–88), higher frequencies of escape mutations in RBM limit their breadth and cross-reactivity in the emerging VOCs (25, 26, 54, 89). For example, the R403K mutation in the class 1 epitope has been classified as a “vaccine escape mutation,” leading to higher infectivity and lower neutralizing activity of vaccine-elicited antibodies against the recent JN.1 subvariant of Omicron (90). Interestingly, the R403D mutation resulted in a 48.9-fold reduction in neutralizing activity of the BCRTH-01 serum, as evidenced in our study. Another identified class 2 epitope residue substitution, L452R, was associated with a massive expansion and immune escape of the Delta variant from vaccine-elicited class 1 and class 2 antibodies (45) during the second year of the pandemic (91). Similarly, the F456L mutation (class 1 epitope) is associated with heightened ACE2 binding, infectivity, and immune evasion from the XBB.1.5 neutralizing antibodies (92), leading to the global spread of the EG.5 (Eris) subvariant (93). The K356T mutation in JN.1, in the class 3 epitope, drives resistance toward Sotrovimab, a class 3 antibody (S309)-based antiviral drug (94). In contrast to classes 1–3, the class 4 epitopes targeted by the selected sera described above show high conservation (Fig. 6C). These class 4 epitopes are situated in the RBD core region, away from the RBM, except for the G416, which resides within RBM.

A major fraction (11/26) of the epitope residues identified in the present study belong to a recently identified epitope (46, 47, 95), rarely seen in the repertoire of antibodies from convalescent sera (42–45). Recently, this epitope has been categorized as class 5 or the RBD8 class of epitopes (27, 31). These antibodies are reported to get enriched by breakthrough infections caused by Omicron subvariants (61). The amino acid residues constituting these epitopes are clustered together at the opposite surface from the ACE2 binding motif of RBD and remain inaccessible in the closed trimeric form of the spike (S) (Fig. 6B). Such epitopes can only be accessible through breathing of the prefusion structure of the trimeric S protein (Fig. 6B), whereby antibody binding ultimately leads to shedding of the S1 subunit (31). Additionally, it has been proposed that the binding of class 5 antibodies leads to premature, receptor-independent conversion of S from pre- to post-fusion conformation, ultimately blocking viral entry and syncytium formation (53). Interestingly, class 5 epitope residues remain completely conserved across all SARS-CoV-2 variants and for a number of different sarbecoviruses (Fig. S5), indicating the cross-reactivity and increased breadth of class 5 antibodies as also observed in our study. It is important to mention that the spike antigen in the ChAdOx vaccine did not contain the two stabilizing proline mutations present in many other recombinant vaccines, including the mRNA-LNP and spike subunit protein vaccines (96–98). A recent study has shown that spike proline stabilization is not required for eliciting neutralizing antibodies, and broadly neutralizing antibodies capable of neutralizing the XBB variant and multiple sarbecoviruses were isolated from non-stabilized spike immunized animals (99). Even though proline stabilization locks the spike in its prefusion conformation, it does not affect the RBD conformation and the accessibility of the inner face epitopes (16, 27). However, it is not currently known whether, *in vivo*, the WT spike in the viral context exhibits greater conformational flexibility and exposure of class 5 epitopes or not. It would therefore be of interest to carry out epitope mapping studies in animals immunized with both stabilized and non-stabilized spikes to address this issue.

Lately, antibodies targeting non-RBM epitopes in the spike protein have emerged as prime candidates for developing broad-spectrum vaccines or therapeutics against SARS-CoV-2 variants (28, 100, 101) and against future emerging SARS-like coronaviruses. However, non-RBM antibodies are limited by their weak neutralization activity. In this study, we show that an immune response elicited by hybrid immunity and containing a combination of class 1 (RBM targeting) and class 5/RBD8 antibodies can achieve high neutralization potencies against different SARS-CoV-2 variants including WA.1, Kappa, Delta, and multiple Omicron subvariants including the more distant XBB.1.5 variant. Characterizing such responses is important for the design and optimization of novel pan sarbecovirus vaccine candidates. Interestingly, the mutation of a single class 5/RBD8 epitope residue (R466D) resulted in a 132-fold reduction in the neutralization titer, which is even higher than that observed in the case of mutations of class 1 (R403D) epitope (48.9-fold), suggesting high abundance and comparable neutralization efficacy of these two different classes of antibodies (class 5/RBD8 and class 1) in one of the selected serum samples (BCRTH-01) used for epitope mapping analysis.

In conclusion, antibodies targeting different classes of epitopes may synergistically contribute to achieving high neutralizing activity (class 1 + 2 epitopes) and broader cross-reactivity (class 4 and RBD8) against all variants of SARS-CoV-2. Notably,class 5/ RBD8 constitutes a significant portion of the epitopes identified in all three serum samples, which differs from the typical composition of the convalescent serum samples analyzed previously (42–45). In India, after the Delta wave in mid-2021, there was little masking or isolation, and likely continued extensive viral exposure. This, coupled with the relatively young population (median age 28.4 years), may have contributed to the generation of broadly cross-reactive antibodies targeting novel cryptic epitope presented here. This warrants further confirmation in larger epitope mapping studies where the humoral immune response and abundance of specific classes of antibodies can be correlated with clinical manifestations of the disease. Greater understanding of this adaptive strategy implemented by the host would pave the way toward the development of universal vaccines and therapeutics against newly emerging SARS-CoV-2 variants and for future sarbecovirus outbreaks.

## MATERIALS AND METHODS

### Cell line

Human embryonic kidney (HEK) 293T cells (ATCC #CRL-3216) were maintained in Dulbecco’s modified Eagle’s medium (DMEM) supplemented with 10% fetal bovine serum (FBS) along with antibiotics like penicillin, streptomycin, and anti-mycotic agent (Gibco) at 37°C and 5% CO_2_. Raji cells, a human B lymphoblastoid cell line, were maintained in RPMI along with 10% FBS.

### Plasmids

Plasmid pHAGE2-EF1aInt-ACE2-WT, expressing human ACE2 gene (BEI#NR-52514), was used for overexpressing ACE2 in HEK 293T cells. Plasmid HDM-IDTSpike-fixK (BEI#NR-52514) was used for expressing the codon-optimized spike protein from SARS-CoV-2, Wuhan-Hu-1 strain (GenBank NC_045512). Plasmids pcDNA3.3-SARS2-B.1.617.1, expressing the spike protein of the Kappa variant (Addgene #172319); pcDNA3.3-SARS2-B.1.617.2 SARS-CoV-2, expressing the spike protein of the Delta variant (Addgene #172320) (102); and SARS-CoV-2 Omicron strain spike gene in Human codon_pcDNA3.1(+), expressing the spike protein of the Omicron variant (B.1.1.529) (GenScript# MC_0101274), were utilized in the study (35). The XBB1.5 spike expression plasmid construct was a kind gift from Dr. Rajesh P. Ringe, Institute of Microbial Technology, Council of Scientific and Industrial Research (CSIR), India.

### Antibodies

NB100-56578 is a polyclonal antibody raised in rabbits against SARS CoV-2 spike protein and was obtained from Novus Biologicals (USA). Serum from the pre-COVID era (single donor human serum off the clot, ISERS2M-2019-33108-07) was obtained from Innovative Research, Inc.

### Human serum collection

Around 164 individuals who had been administered at least two doses of the ChAdOx1 nCoV-19 viral vector vaccine between April and November 2021 with a history of being subsequently infected by the SARS-CoV-2 virus between November 2021 and January 2022 were considered for the study. Serum was extracted from the whole blood of the participants at three different hospitals in the eastern part of India under the supervision of able medical personnel. Following a second written informed consent process, a second lot of whole blood was collected again from nine eligible participants, 2–4 weeks after the first batch of blood collection. Serum was extracted from whole blood and used for the study. Serum recovered from each donor was transferred into a separately labeled tube, aliquoted, and stored at −80°C.

For the ADCC study, whole blood was collected from a healthy donor. PBMCs were isolated and utilized immediately for the experiment.

### Bioinformatics and structural analysis

Genome sequences of 47 different SARS-CoV-2 viruses that infect humans and 11 different sarbecovirus spike RBD sequences were obtained from the GISAID database. Multiple sequence alignment was performed in Bioedit software using the ClustalW multiple alignment function. Further representation of the spike proteins’ aligned RBD amino acid sequences was performed using JALVIEW or the Multlin sequence alignment tool. Structural alignment was performed in Pymol using specific Protein Data Bank (PDB) files of post-fusion spike protein structures of SARS-CoV-2 viruses (PDB ID: 6M0J).

### Construction of SARS-CoV-2 S mutant plasmid constructs

Plasmid HDM-IDTSpike-fixK expressing a codon-optimized spike protein from SARS-CoV-2 was subjected to site-directed mutagenesis to generate R403D, G416D, T430D, and R466D, respectively, using the QuikChange II Site-directed mutagenesis kit (Agilent) according to the manufacturer’s instructions. Mutations generated in the SARS-CoV-2 S protein were further verified using Sanger sequencing.

### Generation of SARS-CoV-2 S pseudotyped lentiviruses

Generation of SARS-CoV-2 lentivirus particles pseudotyped with either full-length WT, different VOCs, or mutation at SARS-CoV-2 spike protein (R403D, G416D, T430D, and R466D) were generated by transfecting HEK293T cells with the respective set of plasmids using the protocol as described previously in reference 103. Briefly, a lentiviral backbone plasmid, pHAGE-CMV-Luc2-IRES-ZsGreen-W (BEI #NR-52516), bearing a CMV promoter to express luciferase, was co-transfected with a set of other lentiviral plasmids like pHDM-Hgpm2, expressing the HIV-1 gag and pol (BEI #NR-52517); pRC-CMV-rev1b, expressing the HIV-1 rev (BEI #NR-52519); pHDM-tat1b, expressing the HIV-1 tat (BEI #NR-52518); and respective different WT or mutant spike protein-expressing plasmids. Posttransfection, the SARS CoV-2 pseudoviruses were collected by passing the supernatant through a 0.45 μm filter and were freshly used.

### Pseudovirus-based serum neutralization assay

Serum samples collected from the volunteers were inactivated at 65°C for 30 minutes prior to use. Serially diluted serum samples were incubated with either WT or different VOC/mutant pseudoviruses at 37°C for 1 hour. Serum-neutralized viral inoculum was then used to infect hACE2-expressing HEK 293T cells at 37°C for 1 hour in the CO_2_ incubator in the presence of polybrene until 18 hours. Successful neutralization of the pseudovirus results in a several-fold reduction in reporter virus activity, which was measured using a multimodal plate reader (GloMax Explorer Multimode Microplate Reader, Promega). Polyclonal SARS CoV-2 (NB100-56578) antibody served as a positive control, whereas sera from the pre-COVID era were used as a negative control for the study.

### Charged scanning library construction and yeast surface display

Substitutions to aspartate and arginine were chosen for introduction in scanning mutagenesis, as analysis of existing deep mutational scanning (DMS) data suggests that these are the most disruptive for binding. A charged scanning library of SARS CoV-2 spike RBD was generated using site-directed mutagenesis. Exposed positions with accessibility >15% (total 125 residues) were individually mutated to aspartate or arginine (for negatively charged residues), and each mutant was fused by PCR to a unique six-nucleotide barcode of known sequence to simplify analysis and eliminate the need for long-read sequencing. Upon mutagenesis by overlap PCR, all the PCR products from each individual mutated position were pooled and transformed into yeast via two-fragment recombination by electroporation.

### Human serum screening

Three human sera were selected for screening based on their high neutralization titers. The sera were first titrated on yeast surface displaying WT-RBD, and a saturating concentration was chosen from the titration curve to be used for epitope mapping. The binding of these sera with the aspartate library was analyzed by flow cytometry, sorted into multiple gates, and then subjected to deep sequencing. Briefly, the pooled library of yeast cells expressing each of the charged mutants was inoculated in 5 mL of SDCAA medium (20 g/L dextrose, 6.7 g/L Difco yeast nitrogen base without amino acids, 5 g/L Bacto casamino acids, 14.7 g/L sodium citrate dihydrate, and 4.29 g/L citric acid monohydrate) and grown overnight at 30°C, 250 rpm. The next day, a secondary inoculation from the overnight culture of the library was grown at 30°C until an OD600 of 3–4, followed by the induction in SGCAA medium (20 g/L galactose, 6.7 g/L Difco yeast nitrogen base without amino acids, 5 g/L Bacto casamino acids, 14.7 g/L sodium citrate dihydrate, and 4.29 g/L citric acid monohydrate) at 20°C for 16 hours. A total of 1 × 10^7^ cells of this library were washed twice with FACS buffer containing 1× phosphate-buffered saline (PBS), pH 7.4 and 0.5% bovine serum albumin (BSA) and then incubated at 4°C for 1 hour with a selected dilution of sera to probe for binding and chicken anti-CMyc antibody (1:300 dilution) as a probe for the expression. The cells were washed twice with FACS buffer and subsequently labeled with Alexa Fluor 633 goat anti-human (1:1,200 dilution) (catalogue no. A21091), plus Alexa Fluor 488 goat anti-chicken (1:300 dilution) (catalogue no. A11039) for 20 minutes at room temperature in the dark. After washing thrice with FACS buffer, cells were resuspended in 1 mL of FACS buffer, subjected to flow cytometry, and sorted on an Aria-III cell sorter (BD Biosciences).

### Deep sequencing and analysis

The sorted yeast cells from each gate were grown separately in liquid SDCAA medium to saturation at 30°C for 24 hours, and the plasmids were extracted using the EZ Yeast plasmid miniprep kit (G Biosciences) according to the manufacturer’s instructions and then subjected to PCR. The PCR primers were designed to amplify the barcode region only (150 bp) and in such a way that for both the forward and reverse end reads, the first three bases are NNN, followed by six bases of unique sequence tag (multiplex identifier, MID) and the 21 bases of the primer sequence complementary to the gene. Each forward MID sequence represents a particular serum, while each reverse MID sequence represents a specific gate. The PCR for all the gates from three sorted sera samples was carried out using Phusion polymerase for 15 cycles. Following agarose gel electrophoresis and gel band purification, an equal amount (∼100 ng) of PCR product from each sample was pooled and then gel-band purified, followed by sequencing on the NovaSeq platform. The fraction of each mutant (*Xi*) distributed across all the bins was calculated as in (equation 1), and then the reconstructed MFI for each mutant was calculated by the summation of the product obtained from multiplying the fraction of a particular mutant in a particular gate (*Xi*) with the MFI of that gate that was obtained from the FACS experiment (*Fi*) (equation 2).


(1) $$Fraction\;of\;mutant\;in\;each\;gate\;{𝑋}_{𝑖}=\frac{Ni}{\sum_{1}^{n}Ni}$$

*Ni*, the number of reads of a specific mutant in bin *i*.


(2) $$\text{Reconstructed MFI}=\sum_{1}^{n}X_{i}\times N_{i}$$

The ratio of MFI expression/MFI binding for all positions was represented by a heatmap using GraphPad Prism software. The mutants with a ratio (normalized MFI of expression:normalized MFI of binding) of one standard deviation higher than the mean MFI were considered as epitope residues.

### Antibody-dependent cellular cytotoxicity (ADCC)

Raji cells were electroporated with a plasmid encoding the SARS-CoV-2 spike protein (40 µg DNA) using the BTX 630 electroporation system. PBMCs were freshly isolated from 15 mL of healthy blood through Ficoll-Hypaque density gradient centrifugation and were cultured in RPMI medium supplemented with 10% fetal calf serum (FCS). The spike protein-expressing Raji cells were incubated with either BCRTH-01 serum or pre-COVID-19 sera (diluted 1:10) at 37°C for 1 hour. Following incubation, the Raji cells were co-cultured with PBMCs at a 1:5 ratio for 6 hours. Immunostaining was then performed. The cells were washed, fixed, and analyzed via flow cytometry. The percentage of positive Raji cells was determined using a PE-conjugated CD19 antibody (diluted 1:200), while the percentage of activated NK cells was assessed using a fluorescein isothiocyanate (FITC)-conjugated CD107a antibody (diluted 1:200). Flow cytometric analysis was carried out on a BD LSRFortessa instrument, and data analysis was performed using the FlowJo software (Windows version 10.6.0; Becton, Dickinson and Company, Ashland, OR, USA, 2019).

### Statistical analysis

All experiments were performed in triplicate, and each data were repeated at least three times except for the epitope mapping experiments, which were performed in duplicate. Graphs were plotted using GraphPad Prism 10.1.2 and were represented as mean standard deviations (*n* = 3). Results were compared by performing a two-tailed Student’s *t*-test. Significance is defined as *P* < 0.05, and statistical significance is indicated with an asterisk (*). *P < 0.001 was considered statistically significant.

## Data Availability

The deep sequencing data discussed in the present study have been deposited in the SRA database under accession number SRR31913842.

## SUPPLEMENTAL MATERIAL

Supplemental material jvi.00051-25-s0001.docx Figures S1 to S6 and Table S1.

ASM does not own the copyrights to Supplemental Material that may be linked to, or accessed through, an article. The authors have granted ASM a non-exclusive, world-wide license to publish the Supplemental Material files. Please contact the corresponding author directly for reuse.
