# Supplementary material for: Early 2022 breakthrough infection sera from India target the conserved cryptic class 5 epitope to counteract immune escape by SARS-CoV-2 variants
Source: J Virol. 2025 Mar 26;99(4):e00051-25. doi: 10.1128/jvi.00051-25 (PMC11998512; doi:10.1128/jvi.00051-25)
Supplement: Supplemental material — Figures S1 to S6 and Table S1. [file jvi.00051-25-s0001.docx]

**Supporting information**

**Early 2022 breakthrough infection sera from India target the conserved cryptic class 5 epitope to counteract immune escape by SARS-CoV-2 variants**

Indrani Das Jana^1^, Kawkab Kanjo^2^, Subhanita Roy^1^, Munmun Bhasin^2^, Shatarupa Bhattacharya^3^, Indranath Banerjee^4^, Subhasis Jana^5^, Arjun Chatterjee^6^, Alok Kumar Chakrabarti^7^, Suman Chakraborty^8^, Budhaditya Mukherjee^3^, Raghavan Varadarajan^2*^, Arindam Mondal^1*^

^1^Department of Bioscience and Biotechnology, Indian Institute of Technology Kharagpur, Kharagpur-721302, West Bengal, India

^2^Molecular Biophysics Unit (MBU), Indian Institute of Science, Bengaluru-560012, Karnataka, India

^3^School of Medical Science and Technology, Indian Institute of Technology Kharagpur, Kharagpur-721302, West Bengal, India

^4^B.C. Roy Technology Hospital, Indian Institute of Technology Kharagpur, Kharagpur-721302, West Bengal, India

^5^Purba Medinipur District Hospital, Tamluk-721636, West Bengal, India

^6^Institute of Neuroscience, Kolkata*-*700017, West Bengal, India

^7^Division of Virology, ICMR-National Institute of Cholera and Enteric Diseases, Kolkata-700010, West Bengal, India

^8^Department of Mechanical Engineering, Indian Institute of Technology Kharagpur, Kharagpur-721302, India

**Correspondence*:** [varadar@iisc.ac.in](mailto:varadar@iisc.ac.in) (R.V.), [arindam.mondal@iitkgp.ac.in](mailto:arindam.mondal@iitkgp.ac.in) (A.M.)


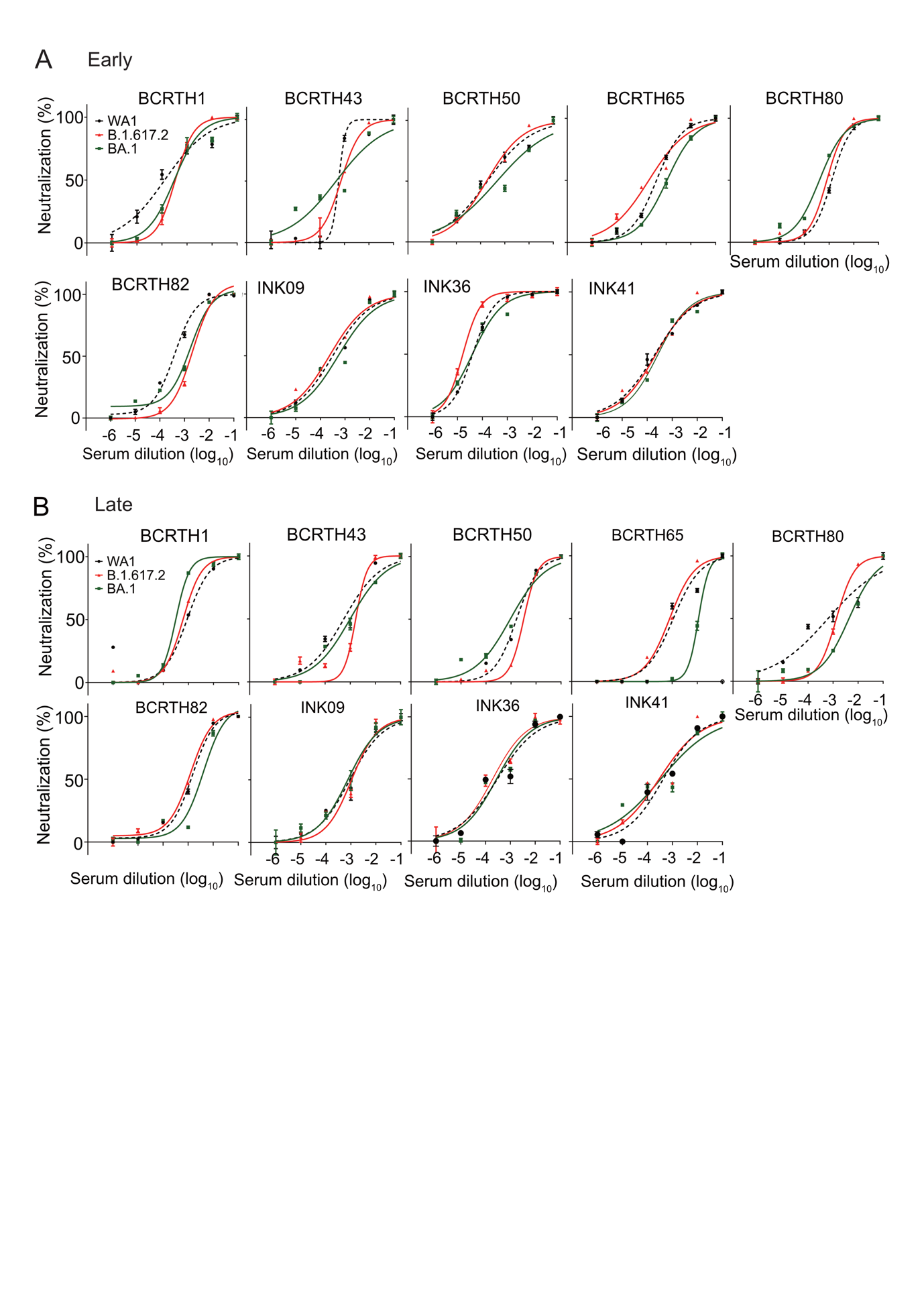


**FIG S1.** Neutralization of the WA.1, B.1.617.2 and BA.1 pseudoviruses with nine serum samples collected from selected volunteers during 1st batch (early) (A) and second batch (late) (B) of sample collection. 100% neutralization of the pseudoviruses is considered to be obtained by the Polyclonal SARS CoV-2 (NB100-56578) antibody. All data shown are averages of the results of at least three independent experiments ± SD.


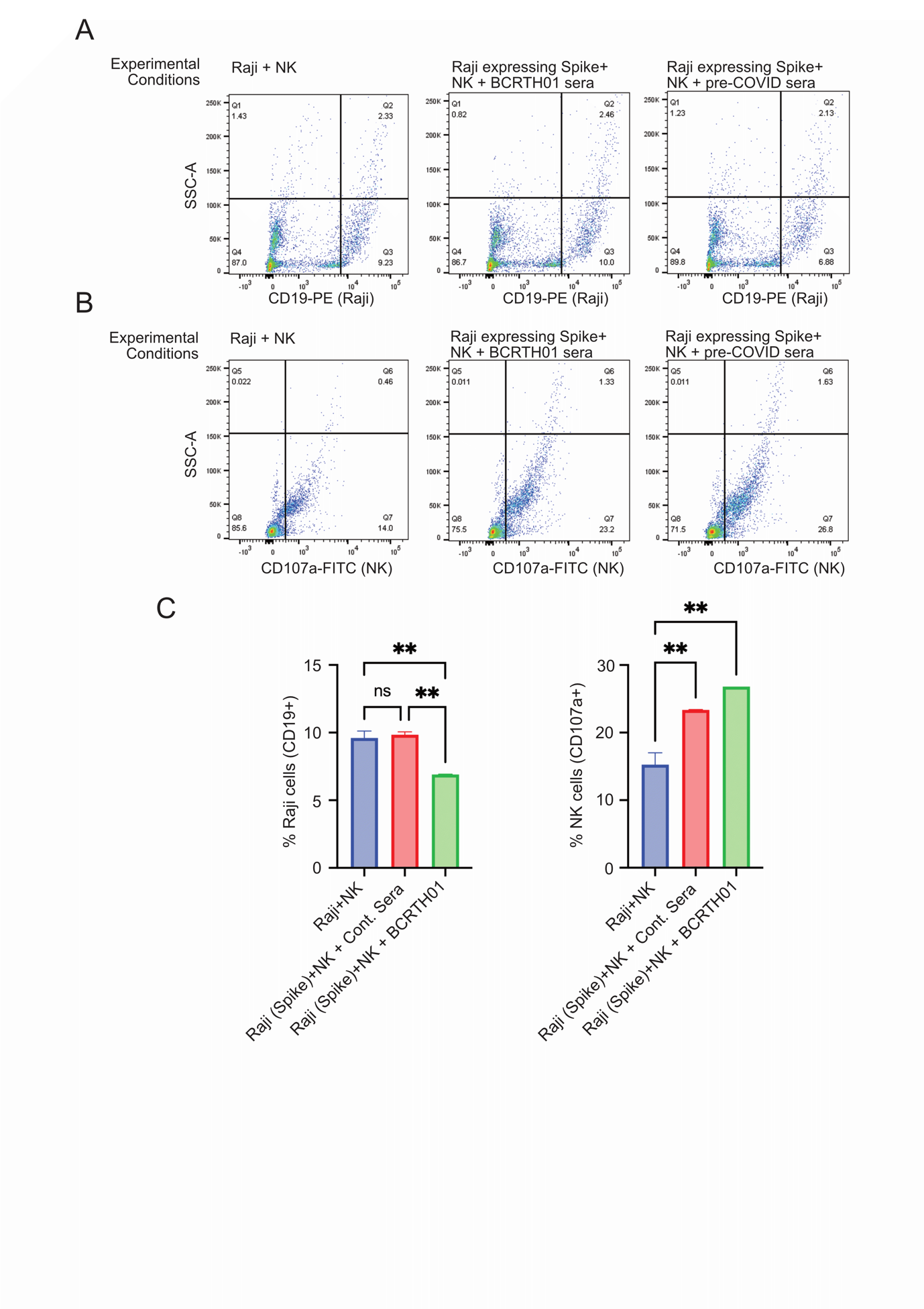


**FIG S2.** Representative flow cytometry images with y-axis representing side scatter (SSC-A) and the x-axis indicating (A) PE-CD19 (Raji cell) or (B) CD107a-FITC (NK cell) under different experimental conditions. (C) Graphical representation of the percentage of (i) Raji cell population and (ii) NK cell population under different experimental conditions. A significant decrease (p=0.0049) in Raji cell population was noticed in response to treatment with BCRTH sera (1:10). (ii) NK cell population under different experimental conditions. A significant increase (P=0.0032) in NK cell population was noticed in response to treatment with BCRTH sera (1:10). Untransfected Raji treated with BCRTH sera (1:10) was kept as control. The experiment is performed in biological triplicate and one-way ANOVA is used to calculate statistical significance.


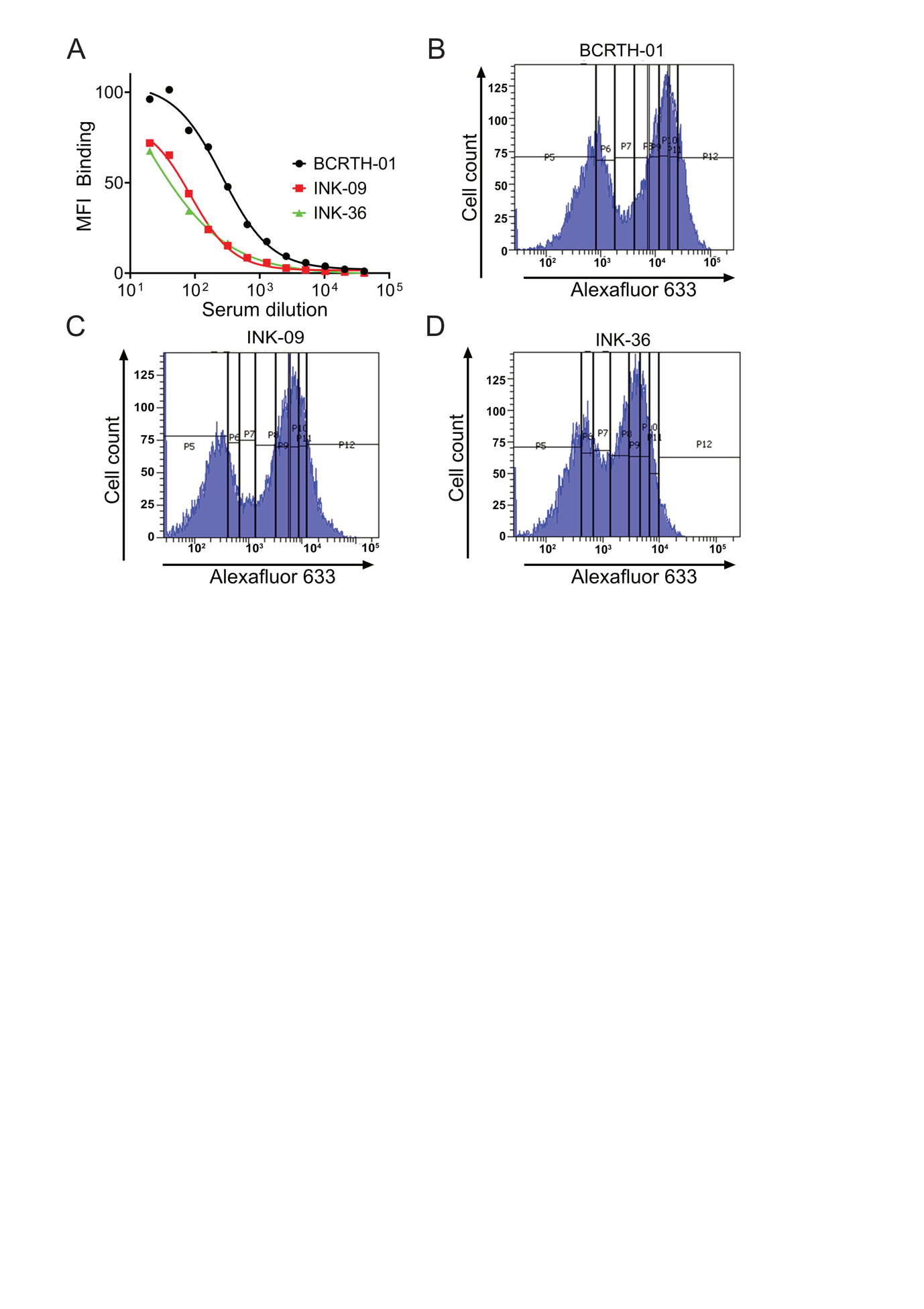


**Fig S3.** (A) The binding profile of the selected serum samples to wild type RBD displayed on the yeast surface. (B-D) Histograms showing yeast cells binding to the indicated serum samples, were sorted into eight gates based upon low to high binding affinity and the frequency of each variant’s barcode in individual gates was determined by Illumina sequencing.


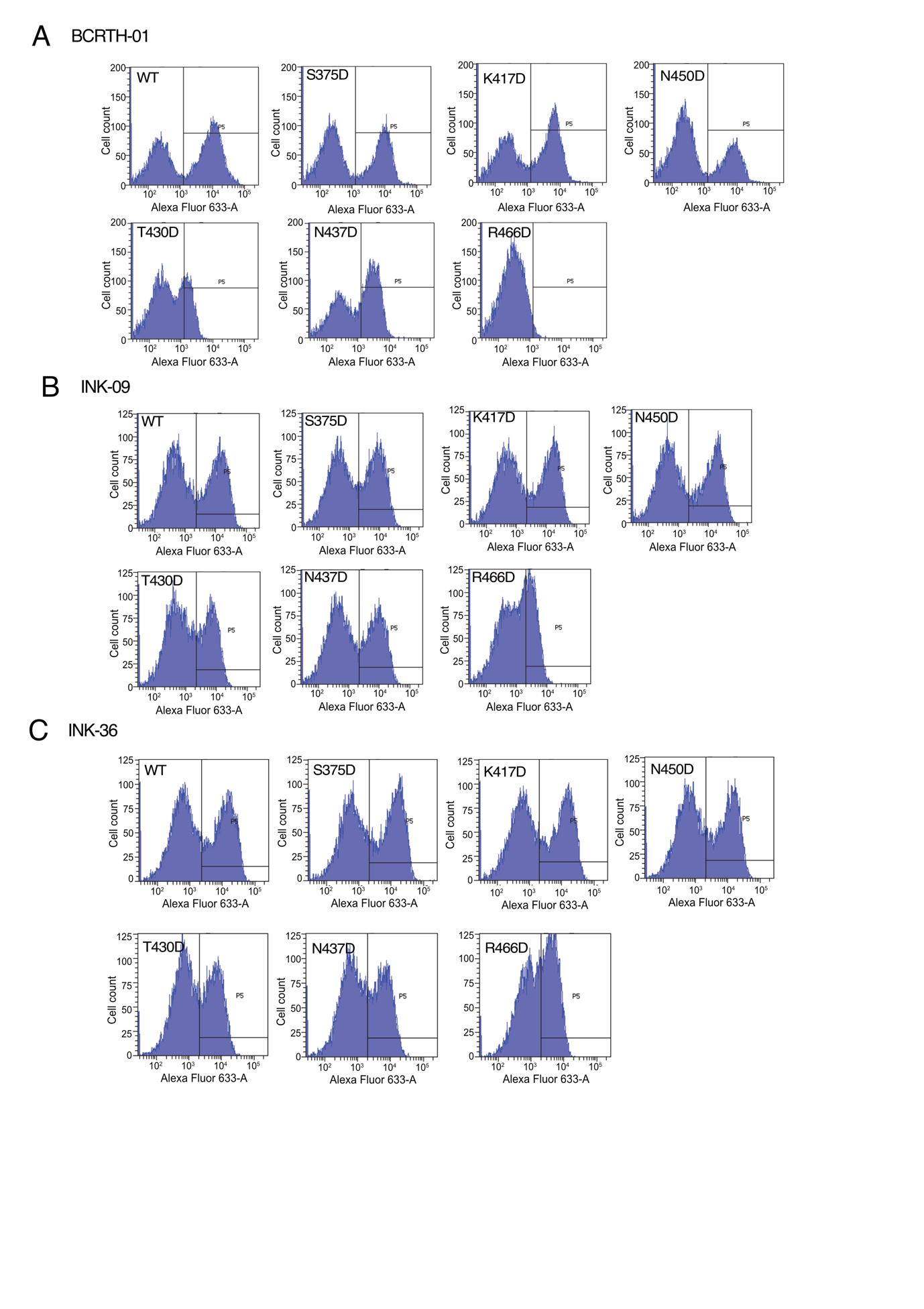
**Fig S4.** Validation of the epitope mapping results was carried out by quantifying the binding of WT and the selected RBD mutants to BCRTH-01, INK09 and INK36 sera (A-C) Histograms showing binding of the yeast cells displaying WT or mutant RBDs to the indicated serum samples, sorted into gates based upon low to high binding. Each image is a representative of two independent experiments performed in triplicates. Non-epitope and epitope residue mutations are shown in top and bottom panels of A-C.

**
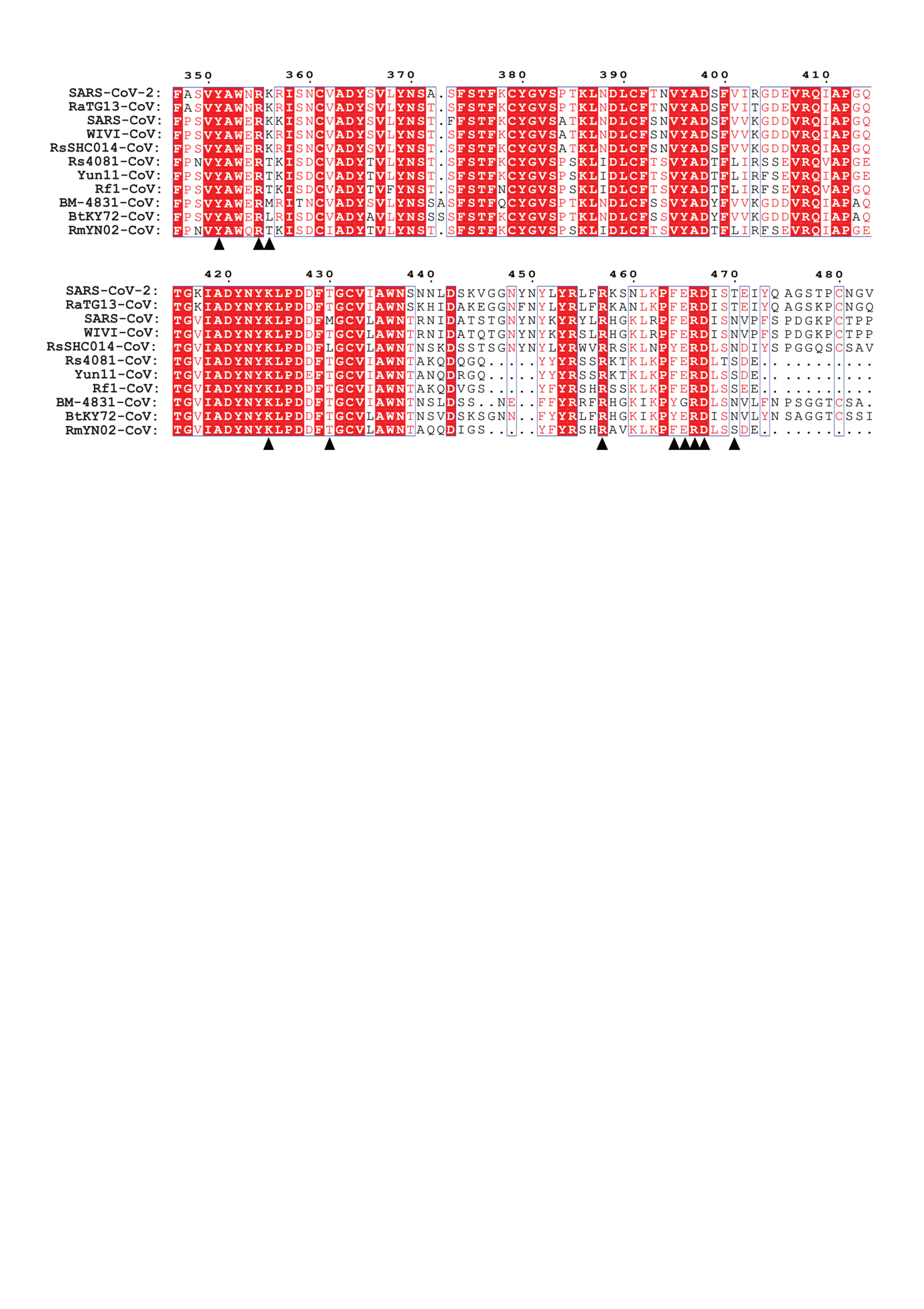
FIG S5.** Multiple sequence alignment of different sarbecovirus spike RBD sequences showing conservation of the Class5/ RBD epitope residues marked by black triangles.


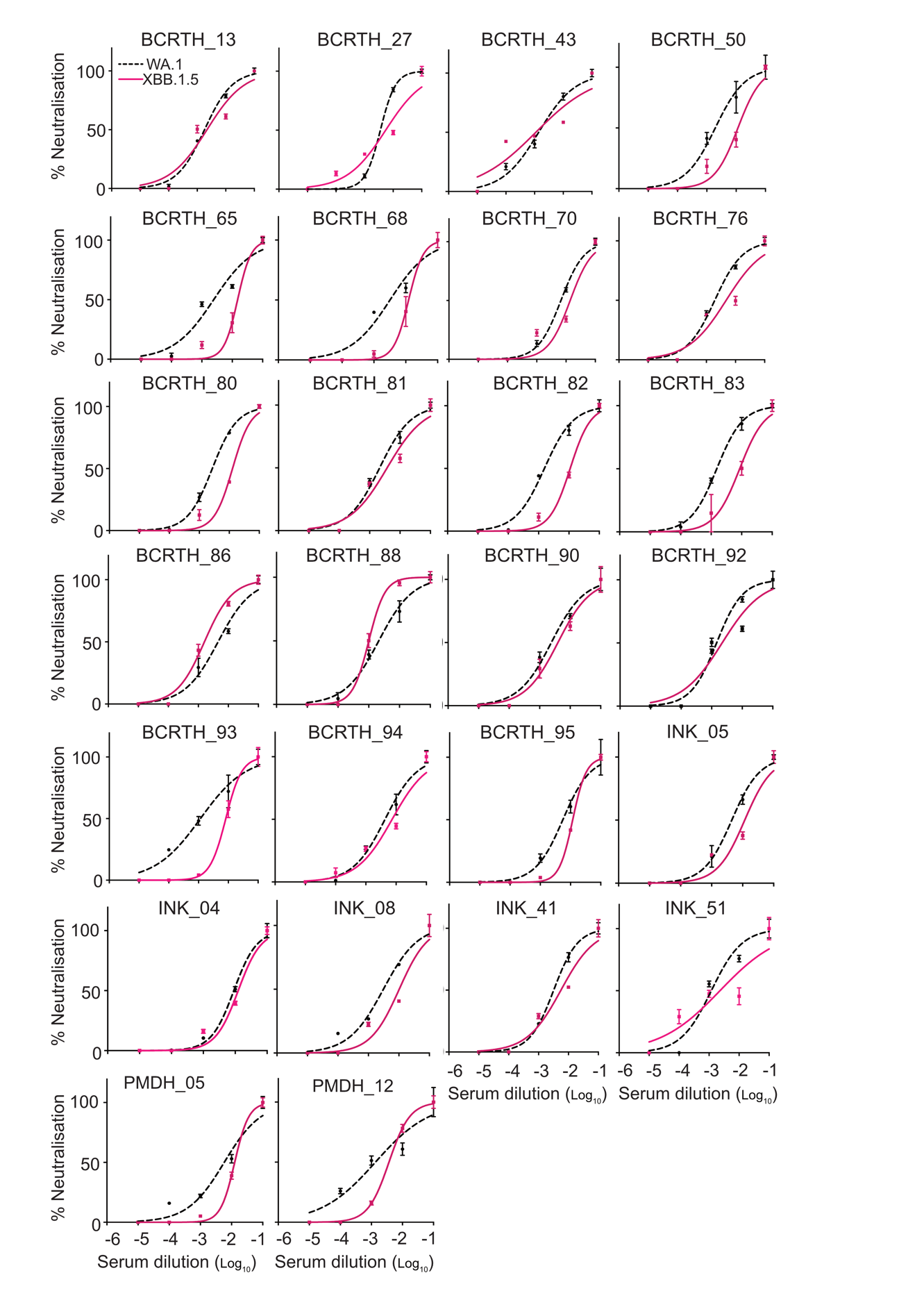


**FIG S6.** Selected 29 sera show neutralising activity against XBB. 1.5. Neutralisation curves for WA. 1 and XBB. 1.5 pseudoviruses. All data shown are averages of the results of at least three independent experiments ± SD.

**Table S1.** **MFI_Ratio_ of all the exposed residues of charged scanning RBD library*.**

|  | **MFI Ratio (BCRTH-01)** | | | **MFI Ratio (INK-09)** | | | **MFI Ratio (INK-36)** | | |
| --- | --- | --- | --- | --- | --- | --- | --- | --- | --- |
| **Mutant** | **Replicate 1** | **Replicate 2** | **Mean** | **Replicate 1** | **Replicate 2** | **Mean** | **Replicate 1** | **Replicate 2** | **Mean** |
| 332 | 0.991 | 1.041 | 1.016 | 1.046 | 1.003 | 1.025 | 0.982 | 0.945 | 0.963 |
| 333 | 1.037 | 1.040 | 1.038 | 1.024 | 1.014 | 1.019 | 0.979 | 0.978 | 0.979 |
| 334 | 1.015 | 1.004 | 1.010 | 0.992 | 0.966 | 0.979 | 0.966 | 0.967 | 0.967 |
| 335 | 1.090 | 1.077 | 1.084 | 1.063 | 1.032 | 1.048 | 1.031 | 1.026 | 1.029 |
| 337 | 1.032 | 1.036 | 1.034 | 0.990 | 0.982 | 0.986 | 1.026 | 0.908 | 0.967 |
| 339 | 1.030 | 1.030 | 1.030 | 1.024 | 0.988 | 1.006 | 1.011 | 0.945 | 0.978 |
| 340 | 1.116 | 1.061 | 1.089 | 1.014 | 1.021 | 1.017 | 0.983 | 0.965 | 0.974 |
| 344 | 1.108 | 1.020 | 1.064 | 0.833 | 0.915 | 0.874 | 1.101 | 0.947 | 1.024 |
| 345 | 0.953 | 1.003 | 0.978 | 0.903 | 0.979 | 0.941 | 0.900 | 0.909 | 0.905 |
| 346 | 1.063 | 1.193 | 1.128 | 1.133 | 1.054 | 1.093 | 1.032 | 0.975 | 1.004 |
| 348 | 1.085 | 1.068 | 1.077 | 1.028 | 1.029 | 1.028 | 1.016 | 1.011 | 1.013 |
| 351 | 1.259 | 1.333 | 1.296 | 1.255 | 1.293 | 1.274 | 1.254 | 1.060 | 1.157 |
| 352 | 1.045 | 1.029 | 1.037 | 0.974 | 1.041 | 1.007 | 0.991 | 1.011 | 1.001 |
| 354 | 1.005 | 1.040 | 1.023 | 0.989 | 0.937 | 0.963 | 0.967 | 0.926 | 0.946 |
| 355 | 1.208 | 1.231 | 1.220 | 1.236 | 1.243 | 1.239 | 1.327 | 1.068 | 1.197 |
| 356 | 1.198 | 1.144 | 1.171 | 1.007 | 1.094 | 1.051 | 1.034 | 0.967 | 1.000 |
| 357 | 0.996 | 1.020 | 1.008 | 0.980 | 1.061 | 1.020 | 0.874 | 1.078 | 0.976 |
| 359 | 1.184 | 1.105 | 1.144 | 1.084 | 1.066 | 1.075 | 1.134 | 1.029 | 1.081 |
| 360 | 1.137 | 1.015 | 1.076 | 1.023 | 1.017 | 1.020 | 0.992 | 0.991 | 0.992 |
| 362 | 1.128 | 1.096 | 1.112 | 1.042 | 1.035 | 1.039 | 1.046 | 0.995 | 1.020 |
| 364 | 1.206 | 1.159 | 1.182 | 1.043 | 1.072 | 1.057 | 1.030 | 0.957 | 0.993 |
| 366 | 0.861 | 0.939 | 0.900 | 0.954 | 0.926 | 0.940 | 0.932 | 0.852 | 0.892 |
| 367 | 0.917 | 0.959 | 0.938 | 0.903 | 0.925 | 0.914 | 0.957 | 0.913 | 0.935 |
| 369 | 1.195 | 1.094 | 1.145 | 1.000 | 1.060 | 1.030 | 1.062 | 0.985 | 1.023 |
| 370 | 1.046 | 1.055 | 1.050 | 1.012 | 0.991 | 1.001 | 1.003 | 0.964 | 0.983 |
| 371 | 0.973 | 1.035 | 1.004 | 1.015 | 0.923 | 0.969 | 1.010 | 0.846 | 0.928 |
| 372 | 1.013 | 1.005 | 1.009 | 0.988 | 0.950 | 0.969 | 1.013 | 0.976 | 0.995 |
| 373 | 1.022 | 1.081 | 1.051 | 1.014 | 1.010 | 1.012 | 1.008 | 0.989 | 0.999 |
| 375 | 0.957 | 0.996 | 0.976 | 1.005 | 0.909 | 0.957 | 0.991 | 0.897 | 0.944 |
| 376 | 1.143 | 1.053 | 1.098 | 1.009 | 1.066 | 1.037 | 0.995 | 0.995 | 0.995 |
| 378 | 1.099 | 1.051 | 1.075 | 1.009 | 0.981 | 0.995 | 0.966 | 0.928 | 0.947 |
| 380 | 1.131 | 1.192 | 1.162 | 1.125 | 1.173 | 1.149 | 1.193 | 0.962 | 1.077 |
| 381 | 1.271 | 1.251 | 1.261 | 1.021 | 1.087 | 1.054 | 1.070 | 0.949 | 1.009 |
| 383 | 0.997 | 1.028 | 1.013 | 0.979 | 0.942 | 0.961 | 0.999 | 0.956 | 0.978 |
| 384 | 1.137 | 1.039 | 1.088 | 1.016 | 1.030 | 1.023 | 0.975 | 0.977 | 0.976 |
| 385 | 1.034 | 1.079 | 1.057 | 1.080 | 0.964 | 1.022 | 1.050 | 1.004 | 1.027 |
| 386 | 1.019 | 1.014 | 1.016 | 1.063 | 0.931 | 0.997 | 0.977 | 0.972 | 0.975 |
| 387 | 1.240 | 1.222 | 1.231 | 1.035 | 1.074 | 1.054 | 1.015 | 0.969 | 0.992 |
| 389 | 1.087 | 1.045 | 1.066 | 1.001 | 0.993 | 0.997 | 0.986 | 0.965 | 0.975 |
| 390 | 1.179 | 1.111 | 1.145 | 0.999 | 1.030 | 1.014 | 0.994 | 0.995 | 0.994 |
| 396 | 1.191 | 1.099 | 1.145 | 0.994 | 1.021 | 1.008 | 1.016 | 0.978 | 0.997 |
| 403 | 1.354 | 1.231 | 1.292 | 1.120 | 1.226 | 1.173 | 1.210 | 1.030 | 1.120 |
| 405 | 1.093 | 1.000 | 1.047 | 1.015 | 0.972 | 0.994 | 0.984 | 0.944 | 0.964 |
| 408 | 0.986 | 1.044 | 1.015 | 1.035 | 0.982 | 1.008 | 0.994 | 0.981 | 0.988 |
| 413 | 1.149 | 1.109 | 1.129 | 1.018 | 1.011 | 1.014 | 0.980 | 0.956 | 0.968 |
| 415 | 1.064 | 0.995 | 1.030 | 0.967 | 0.989 | 0.978 | 0.984 | 0.949 | 0.967 |
| 416 | 1.336 | 1.323 | 1.330 | 1.144 | 1.208 | 1.176 | 1.184 | 1.018 | 1.101 |
| 417 | 0.989 | 0.974 | 0.981 | 0.974 | 0.978 | 0.976 | 0.975 | 0.974 | 0.974 |
| 420 | 1.225 | 1.102 | 1.164 | 0.993 | 0.995 | 0.994 | 1.133 | 1.012 | 1.072 |
| 421 | 1.120 | 1.316 | 1.218 | 1.168 | 1.170 | 1.169 | 1.121 | 1.110 | 1.115 |
| 424 | 1.170 | 1.121 | 1.145 | 1.154 | 1.230 | 1.192 | 1.173 | 0.990 | 1.082 |
| 427 | 1.288 | 0.955 | 1.122 | 1.111 | 0.969 | 1.040 | 1.010 | 0.939 | 0.975 |
| 428 | 1.150 | 1.059 | 1.105 | 0.972 | 1.090 | 1.031 | 1.093 | 1.018 | 1.055 |
| 430 | 1.296 | 1.244 | 1.270 | 1.041 | 1.126 | 1.083 | 1.070 | 0.976 | 1.023 |
| 437 | 1.305 | 1.299 | 1.302 | 1.077 | 1.139 | 1.108 | 1.074 | 1.161 | 1.117 |
| 439 | 1.085 | 1.019 | 1.052 | 0.997 | 0.956 | 0.976 | 1.005 | 0.975 | 0.990 |
| 440 | 0.992 | 1.047 | 1.020 | 0.968 | 0.891 | 0.929 | 0.968 | 0.975 | 0.971 |
| 441 | 1.042 | 1.035 | 1.038 | 1.023 | 1.008 | 1.016 | 1.018 | 0.985 | 1.001 |
| 444 | 1.057 | 1.010 | 1.033 | 1.000 | 0.968 | 0.984 | 1.052 | 0.991 | 1.021 |
| 445 | 1.023 | 1.077 | 1.050 | 1.039 | 1.001 | 1.020 | 0.969 | 1.041 | 1.005 |
| 446 | 1.022 | 1.039 | 1.030 | 1.052 | 1.018 | 1.035 | 1.030 | 1.030 | 1.030 |
| 448 | 1.122 | 1.086 | 1.104 | 1.059 | 0.991 | 1.025 | 1.030 | 1.068 | 1.049 |
| 449 | 1.019 | 1.029 | 1.024 | 1.041 | 0.969 | 1.005 | 1.013 | 1.007 | 1.010 |
| 450 | 0.984 | 1.039 | 1.012 | 0.976 | 0.926 | 0.951 | 0.973 | 1.003 | 0.988 |
| 452 | 1.101 | 1.077 | 1.089 | 1.100 | 1.016 | 1.058 | 1.078 | 1.074 | 1.076 |
| 455 | 0.967 | 1.032 | 0.999 | 1.032 | 0.999 | 1.015 | 1.015 | 0.972 | 0.994 |
| 456 | 1.241 | 1.131 | 1.186 | 1.205 | 1.300 | 1.253 | 1.233 | 1.152 | 1.193 |
| 457 | 1.167 | 1.284 | 1.225 | 1.247 | 1.199 | 1.223 | 1.170 | 0.939 | 1.055 |
| 458 | 1.033 | 0.988 | 1.011 | 1.019 | 0.991 | 1.005 | 1.002 | 0.966 | 0.984 |
| 459 | 0.940 | 0.977 | 0.958 | 1.039 | 0.939 | 0.989 | 0.900 | 0.936 | 0.918 |
| 460 | 1.023 | 0.989 | 1.006 | 0.978 | 0.924 | 0.951 | 0.905 | 1.019 | 0.962 |
| 462 | 1.011 | 0.976 | 0.993 | 1.012 | 0.975 | 0.993 | 0.935 | 0.993 | 0.964 |
| 463 | 1.237 | 1.041 | 1.139 | 0.986 | 1.029 | 1.007 | 1.041 | 0.965 | 1.003 |
| 464 | 1.336 | 1.158 | 1.247 | 1.113 | 1.143 | 1.128 | 1.109 | 1.066 | 1.088 |
| 465 | 1.216 | 1.236 | 1.226 | 0.984 | 1.102 | 1.043 | 1.072 | 1.089 | 1.080 |
| 466 | 1.328 | 1.355 | 1.342 | 1.158 | 1.216 | 1.187 | 1.216 | 1.017 | 1.117 |
| 467 | 1.233 | 1.252 | 1.243 | 1.224 | 1.220 | 1.222 | 1.220 | 1.017 | 1.119 |
| 468 | 1.023 | 1.024 | 1.023 | 1.023 | 0.997 | 1.010 | 0.928 | 1.027 | 0.977 |
| 469 | 1.100 | 1.118 | 1.109 | 1.013 | 1.102 | 1.057 | 1.080 | 1.043 | 1.062 |
| 470 | 1.215 | 1.142 | 1.179 | 1.122 | 1.000 | 1.061 | 1.095 | 1.033 | 1.064 |
| 471 | 0.951 | 0.965 | 0.958 | 0.904 | 0.993 | 0.948 | 0.862 | 0.919 | 0.890 |
| 473 | 1.257 | 1.082 | 1.170 | 1.182 | 1.109 | 1.146 | 1.138 | 1.062 | 1.100 |
| 474 | 1.016 | 0.977 | 0.996 | 1.001 | 0.957 | 0.979 | 0.920 | 0.927 | 0.923 |
| 475 | 1.205 | 0.972 | 1.088 | 1.031 | 1.006 | 1.019 | 1.163 | 1.042 | 1.102 |
| 476 | 1.040 | 1.003 | 1.021 | 0.986 | 1.046 | 1.016 | 0.962 | 1.072 | 1.017 |
| 477 | 1.003 | 0.970 | 0.986 | 0.985 | 0.940 | 0.963 | 0.977 | 0.915 | 0.946 |
| 478 | 1.001 | 1.101 | 1.051 | 0.996 | 0.975 | 0.985 | 1.006 | 0.965 | 0.986 |
| 479 | 1.000 | 1.067 | 1.034 | 1.023 | 0.941 | 0.982 | 0.935 | 0.946 | 0.941 |
| 481 | 1.008 | 1.029 | 1.018 | 0.991 | 0.995 | 0.993 | 1.032 | 0.983 | 1.007 |
| 482 | 1.077 | 1.086 | 1.081 | 0.996 | 1.041 | 1.019 | 0.997 | 1.045 | 1.021 |
| 483 | 1.048 | 1.145 | 1.097 | 1.079 | 1.020 | 1.049 | 1.001 | 0.977 | 0.989 |
| 484 | 1.091 | 1.043 | 1.067 | 1.041 | 1.021 | 1.031 | 0.995 | 0.939 | 0.967 |
| 485 | 1.052 | 1.089 | 1.070 | 1.006 | 0.980 | 0.993 | 1.013 | 1.097 | 1.055 |
| 486 | 0.959 | 1.040 | 0.999 | 1.029 | 1.032 | 1.030 | 0.998 | 0.936 | 0.967 |
| 487 | 1.049 | 0.986 | 1.017 | 0.972 | 1.008 | 0.990 | 1.031 | 1.019 | 1.025 |
| 489 | 1.131 | 1.161 | 1.146 | 1.296 | 1.172 | 1.234 | 1.171 | 1.119 | 1.145 |
| 490 | 1.062 | 1.007 | 1.034 | 1.018 | 0.954 | 0.986 | 1.010 | 1.051 | 1.031 |
| 493 | 1.044 | 1.069 | 1.057 | 1.018 | 1.008 | 1.013 | 1.054 | 0.994 | 1.024 |
| 494 | 1.070 | 1.026 | 1.048 | 1.015 | 1.042 | 1.029 | 1.034 | 1.050 | 1.042 |
| 496 | 0.989 | 1.029 | 1.009 | 1.056 | 0.974 | 1.015 | 1.001 | 0.985 | 0.993 |
| 498 | 0.927 | 1.023 | 0.975 | 1.103 | 0.956 | 1.030 | 1.059 | 0.928 | 0.993 |
| 499 | 1.083 | 1.071 | 1.077 | 1.077 | 1.045 | 1.061 | 1.071 | 1.035 | 1.053 |
| 500 | 0.955 | 1.041 | 0.998 | 1.014 | 0.938 | 0.976 | 1.015 | 0.907 | 0.961 |
| 501 | 1.077 | 1.091 | 1.084 | 1.087 | 1.050 | 1.069 | 1.062 | 1.024 | 1.043 |
| 502 | 0.960 | 1.008 | 0.984 | 0.993 | 0.952 | 0.972 | 1.033 | 0.918 | 0.976 |
| 503 | 0.995 | 1.015 | 1.005 | 0.964 | 0.958 | 0.961 | 0.977 | 0.914 | 0.946 |
| 504 | 0.950 | 1.052 | 1.001 | 1.001 | 0.979 | 0.990 | 1.028 | 0.998 | 1.013 |
| 505 | 0.973 | 1.083 | 1.028 | 1.044 | 0.960 | 1.002 | 0.992 | 0.943 | 0.968 |
| 516 | 1.129 | 1.000 | 1.064 | 0.949 | 1.015 | 0.982 | 0.929 | 0.974 | 0.952 |
| 517 | 1.052 | 0.999 | 1.026 | 0.960 | 0.961 | 0.960 | 0.978 | 0.991 | 0.985 |
| 518 | 0.972 | 1.041 | 1.007 | 1.003 | 0.957 | 0.980 | 0.990 | 0.905 | 0.948 |
| 519 | 0.965 | 0.987 | 0.976 | 0.972 | 0.914 | 0.943 | 0.984 | 0.899 | 0.942 |
| 520 | 0.983 | 0.997 | 0.990 | 0.956 | 0.934 | 0.945 | 0.967 | 0.939 | 0.953 |
| 521 | 1.027 | 0.976 | 1.001 | 1.004 | 0.933 | 0.969 | 0.943 | 0.936 | 0.939 |
| 522 | 1.010 | 1.028 | 1.019 | 0.957 | 0.976 | 0.967 | 0.978 | 0.981 | 0.979 |
| 523 | 0.978 | 0.998 | 0.988 | 0.958 | 0.876 | 0.917 | 1.031 | 0.948 | 0.989 |
| 527 | 1.041 | 1.035 | 1.038 | 0.997 | 0.955 | 0.976 | 1.009 | 0.958 | 0.983 |
| 528 | 0.994 | 1.019 | 1.006 | 0.995 | 0.970 | 0.983 | 0.966 | 0.948 | 0.957 |
| 529 | 0.960 | 0.995 | 0.978 | 0.943 | 0.921 | 0.932 | 0.929 | 0.927 | 0.928 |
| 530 | 0.960 | 0.993 | 0.976 | 0.914 | 0.908 | 0.911 | 0.950 | 0.918 | 0.934 |
| 531 | 0.953 | 0.968 | 0.960 | 0.933 | 0.933 | 0.933 | 0.922 | 0.893 | 0.907 |
| 532 | 0.912 | 0.925 | 0.918 | 0.892 | 0.880 | 0.886 | 0.915 | 0.871 | 0.893 |
| 533 | 1.016 | 1.022 | 1.019 | 0.964 | 0.969 | 0.966 | 0.966 | 0.936 | 0.951 |

*The residues with cut-off values (MFI_Ratio_ one standard deviation (1SD) higher than the mean MFI_Ratio_) were designated as epitope residues. The cut-off values for the mean of two replicates for BCRTH-01, INK-09, and INK-36 are: 1.162, 1.097, and 1.063, respectively.
